# Filter exchange imaging with crusher gradient modelling detects increased blood–brain barrier water permeability in response to mild lung infection

**DOI:** 10.1186/s12987-023-00422-7

**Published:** 2023-04-03

**Authors:** Yolanda Ohene, William J. Harris, Elizabeth Powell, Nina W. Wycech, Katherine F. Smethers, Samo Lasič, Kieron South, Graham Coutts, Andrew Sharp, Catherine B. Lawrence, Hervé Boutin, Geoff J. M. Parker, Laura M. Parkes, Ben R. Dickie

**Affiliations:** 1 Division of Psychology, Communication and Human Neuroscience, School of Health Sciences, Faculty of Biology, Medicine and Health, University of Manchester, Zochonis Building, Oxford Road, Manchester, M13 9PL UK; 2 Geoffrey Jefferson Brain Research Centre, Manchester Academic Health Science Centre, University of Manchester, Manchester, UK; 3 Division of Neuroscience, School of Biological Sciences, Faculty of Biology, Medicine and Health, University of Manchester, Manchester, UK; 4 Centre for Medical Image Computing, Department of Medical Physics and Biomedical Engineering and Department of Neuroinflammation, UCL, London, UK; 5 Danish Research Centre for Magnetic Resonance, Centre for Functional and Diagnostic Imaging and Research, Copenhagen University Hospital Amager and Hvidovre, Copenhagen, Denmark; 6 Random Walk Imaging, Lund, Sweden; 7 Evotec (UK) Ltd., Alderley Park, Block 23F, Mereside, Cheshire, SK10 4TG UK; 8 Bioxydyn Limited, Manchester, UK; 9 Division of Informatics, Imaging and Data Sciences, Faculty of Biology, Medicine and Health, University of Manchester, Manchester, UK

**Keywords:** MRI, FEXI, Blood–brain barrier, BBB water permeability, BBB water exchange, Infection, Aquaporin-4

## Abstract

Blood–brain barrier (BBB) dysfunction occurs in many brain diseases, and there is increasing evidence to suggest that it is an early process in dementia which may be exacerbated by peripheral infection. Filter-exchange imaging (FEXI) is an MRI technique for measuring trans-membrane water exchange. FEXI data is typically analysed using the apparent exchange rate (AXR) model, yielding estimates of the AXR. Crusher gradients are commonly used to remove unwanted coherence pathways arising from longitudinal storage pulses during the mixing period. We first demonstrate that when using thin slices, as is needed for imaging the rodent brain, crusher gradients result in underestimation of the AXR. To address this, we propose an extended crusher-compensated exchange rate (CCXR) model to account for diffusion-weighting introduced by the crusher gradients, which is able to recover ground truth values of BBB water exchange (*k*_in_) in simulated data. When applied to the rat brain, *k*_in_ estimates obtained using the CCXR model were 3.10 s^−1^ and 3.49 s^−1^ compared to AXR estimates of 1.24 s^−1^ and 0.49 s^−1^ for slice thicknesses of 4.0 mm and 2.5 mm respectively. We then validated our approach using a clinically relevant *Streptococcus pneumoniae* lung infection. We observed a significant 70 ± 10% increase in BBB water exchange in rats during active infection (*k*_in_ = 3.78 ± 0.42 s^−1^) compared to before infection (*k*_in_ = 2.72 ± 0.30 s^−1^; p = 0.02). The BBB water exchange rate during infection was associated with higher levels of plasma von Willebrand factor (VWF), a marker of acute vascular inflammation. We also observed 42% higher expression of perivascular aquaporin-4 (AQP4) in infected animals compared to non-infected controls, while levels of tight junction proteins remain consistent between groups. In summary, we propose a modelling approach for FEXI data which removes the bias in estimated water-exchange rates associated with the use of crusher gradients. Using this approach, we demonstrate the impact of peripheral infection on BBB water exchange, which appears to be mediated by endothelial dysfunction and associated with an increase in perivascular AQP4.

**Supplementary Information:**

The online version contains supplementary material available at 10.1186/s12987-023-00422-7.

## Introduction

The blood–brain barrier (BBB) is a vital component of the neurovascular unit (NVU) responsible for protecting the brain from harmful toxins and pathogens present in the bloodstream, while also enabling selective passage of essential nutrients and molecules from the bloodstream into the brain. Emerging evidence suggests that BBB dysfunction occurs in early Alzheimer’s Disease (AD) [1, 2], possibly through interaction between the BBB and neuro-inflammatory mediators within the brain (e.g. β-amyloid, tau) [3–5], or from interaction with systemic inflammatory factors (e.g. resulting from peripheral infection) [6]. Peripheral infection in dementia patients typically leads to delirium, a syndrome that may arise due to exacerbation of an already compromised BBB [7]. However, there is a gap in understanding of how peripheral infection affects the BBB, and the compounding impact that it may have in initiating or worsening neurodegenerative pathology. While impairment of the BBB in early stage dementia is low-level, causing only minimal leakage of gadolinium-based contrast agents (GBCA) [1, 8], it has been shown to have important consequences for cognition [9, 10]. Highly sensitive measurement techniques are now needed to study these early subtle BBB impairments, and to understand how peripheral infection may compound or exacerbate these alterations.

Dynamic contrast enhanced magnetic resonance imaging (DCE-MRI) is a commonly used imaging tool for measuring BBB permeability, but it lacks sensitivity and reliability when probing subtle BBB dysfunction [11–13]. Advanced contrast-based methods have been developed to measure BBB water-exchange based on shortening blood T1 [14–17]. Using these methods, we have detected elevated BBB water exchange in a transgenic rat model of AD (TgF344-AD) compared to age-matched wild-types. We also observed increases in water-exchange due to ageing in both groups but found these subtle BBB changes occurred earlier in AD rats compared to wild-types [16, 17]. Importantly, measurements of BBB integrity made using standard DCE-MRI in the same animals did not show differences between groups. Water is a far smaller molecule compared to GBCAs (18 Da versus 400–800 Da), therefore techniques that probe water permeability have the potential to detect earlier changes, or different types of BBB damage, such as non-disruptive or diffuse changes, which could provide additional understanding about neuroinflammation and neurodegenerative pathologies. A further contrast-based MRI approach (contrast-enhanced arterial spin labelling (ASL)) is able to measure BBB water exchange in the human brain [18], and can be used with lower contrast doses [19].

In recent years, several advanced non-invasive MRI techniques have emerged, commonly based on ASL [20–26] or diffusion-encoding MRI methods [27, 28], that are able to measure BBB water exchange without the use of contrast agents. Eliminating the contrast agent removes the need for intravenous injections, allows those with renal problems to be scanned and avoids potential problems associated with contrast agent accumulation [29]. Using aquaporin-4 (*Aqp4*)-deficient mice, we previously showed multiple-echo time ASL is sensitive to changes in BBB water permeability caused by reduced expression of astrocytic AQP4 [30], and also found increases in water-permeability with ageing [31], agreeing with results from Dickie et al*.* using contrast-based methods [17]. Other ASL techniques, such as diffusion-prepared ASL and WEPCAST, have also been able to detect differences in BBB water exchange within several patient groups including those with mild cognitive impairment (MCI) [32], and vascular risk factors [33].

**Fig. 1:**
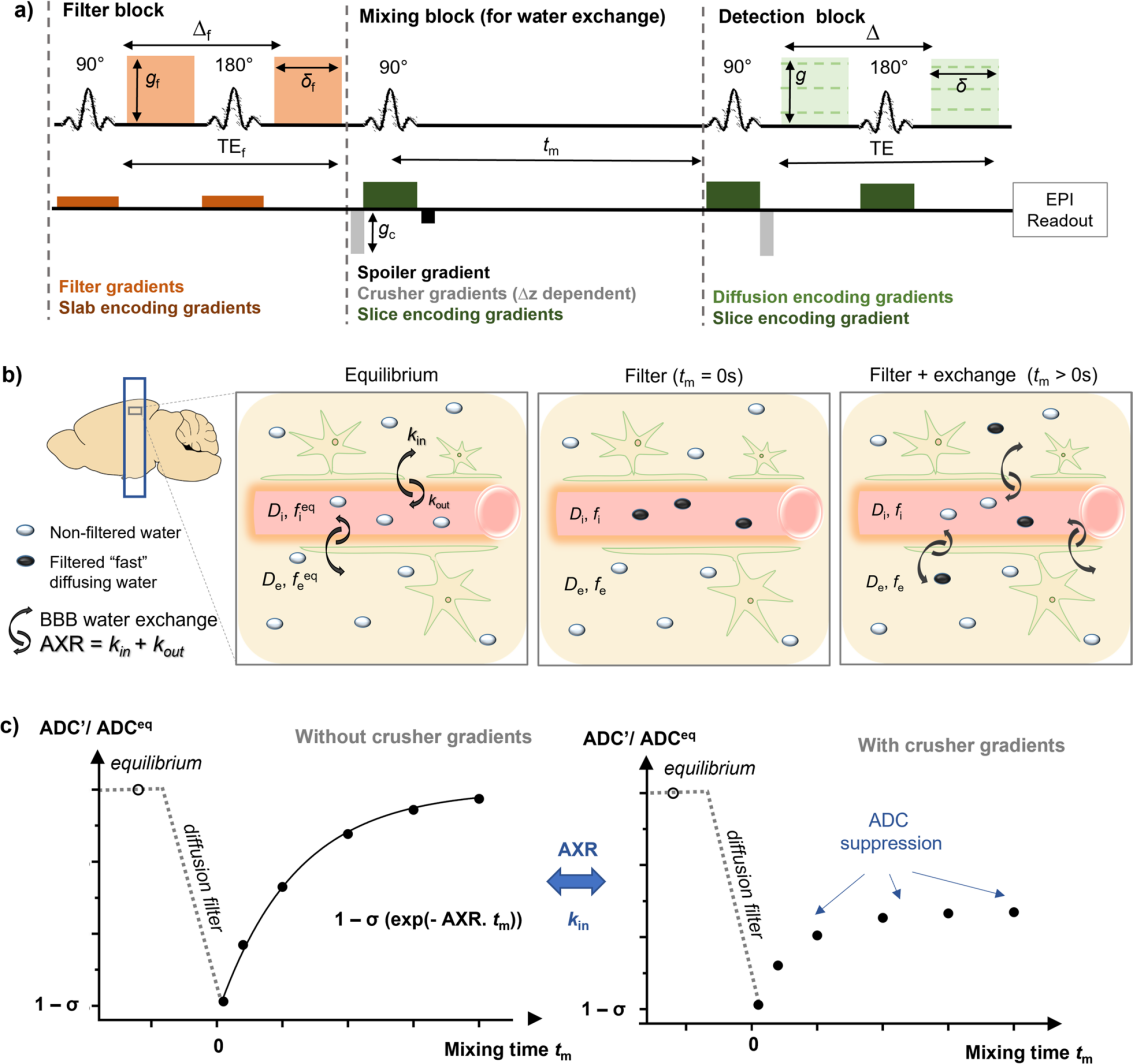
Imaging BBB water exchange. **a** FEXI pulse sequence diagram. The filter block consists of a 90° excitation pulse with associated slab-selection gradient (dark orange), followed by a fixed pair of diffusion-encoding gradients (with filter gradient amplitude (*g*_f_), duration (*δ*_f_) and diffusion time (*∆*_f_), light orange) separated by a slab selective refocussing pulse. The mixing block, for encoding water exchange during varying mixing time *t*_m_, consists of crusher gradients (grey) (with crusher gradient amplitude (*g*_c_) dependent on slice thickness (∆*z*)), slice encoding gradients (dark green) associated with the RF pulses, and a spoiler gradient (black) to null unwanted transverse magnetization. The detection block, for signal readout, consists of variable diffusion encoding gradients (with gradient amplitude (*g*), duration (*δ*) and diffusion time (*∆*), light green) followed by an echo planar imaging (EPI) readout. Adapted from:[43]. **b**, **c** AXR model for water exchange across BBB. A two-compartment model of water-exchange can be parameterised by forward/ backwards exchange rates (*k*_in_ / *k*_out_), intra/extra-vascular diffusivities (*D*_i_/ *D*_e_) and intra/extra-vascular signal fractions (*f*_i_/ *f*_e_). Measurements of the ADC are first acquired at equilibrium (ADC^eq^) without the diffusion filter. The diffusion filter is then applied, which supresses the fast-diffusing intravascular water, leading to a reduction in the measured ADC (ADC'). As the mixing time increases, water-exchange between the two compartments results in a recovery of the ADC’ back to ADC^eq^. The crusher gradients (higher magnitude with smaller ∆*z*) introduce additional diffusion-weighting that results in an undesirable suppression of the ADC'(*t*_m_) recovery. The apparent exchange rate (AXR) models the rate of ADC' recovery as an exponential. Since this model does not account for diffusion weighting caused by the longitudinal storage crusher gradients, it underestimates the true exchange rate when these crusher gradients contribute significantly to the overall diffusion weighting of the filter block

Filter-exchange imaging (FEXI) is a further non-invasive technique that utilises differences in compartmental diffusivities to measure water exchange processes [34, 35]. FEXI consists of two diffusion encoding blocks. The first block acts as a diffusion filter and suppresses the signal component pertaining to the fast-diffusing compartment leading to an apparent reduction in the diffusivity. The second block measures the apparent diffusivity after a variable mixing time, which allows recovery of the diffusivity back to equilibrium dependent on how fast water is exchanging between the compartments. The rate of recovery, called the apparent exchange rate (AXR), can be quantified as an index of water exchange [35], Fig. 1. FEXI has been able to discriminate between transcellular water exchange in brain tumours and their subtypes [35, 36], breast tumours [37] and detect the presence of urea transporters [38]. FEXI has recently been adapted, by using a diffusion filter with a low b-value, to target the intravascular space (taking advantage of the 10–100-fold difference in intravascular and extravascular diffusivities) to measure water exchange across the BBB in the human brain [27, 39]. FEXI has an advantage compared to ASL-based MRI techniques; since it is not based on arterial spin tagging, estimation of BBB water exchange does not require estimation of arterial or other pre-exchange transit times. The adapted FEXI method, here termed “BBB-FEXI”, has been applied to brain tumours [40] and AD patients [41] but has yet to be used to examine subtle BBB pathology due to infection.

While BBB-FEXI has shown promise in the human brain [27, 39, 42], developing this method in the rodent brain comes with an additional challenge: the markedly smaller brain size requires thinner imaging slices. The size of the imaging slice determines the magnitude of the crusher imaging gradients, an essential part of the FEXI MRI sequence as proposed by Nilsson et al., that are needed for selecting the correct coherence pathways for the MRI signal [35]. Previously, Lasič et al*.* demonstrated that high crusher gradient magnitudes cause an underestimation of the water exchange rate using standard AXR modelling [43]. These effects are exacerbated when using low filter b-values for the diffusion filter and biases are greatest for low exchange rates. Therefore, there is a need to explore extended signal models that incorporate the effects of the crusher gradients, facilitating the use of thinner imaging slices needed in the rodent brain.

In this work, we aim to establish a reliable BBB-FEXI method to probe BBB water permeability in the rat brain and test its sensitivity to detect BBB alterations caused by *Streptococcus pneumoniae* lung infection. We propose a more general crusher-compensated exchange rate (CCXR) model that directly describes the effect of crusher gradients on the signal, thereby removing the bias in the estimated water exchange rate. We conduct four studies to evaluate our proposed CCXR model in comparison with the existing AXR model. In study 1, we evaluate the impact of the crusher gradients on the recovery of apparent diffusion coefficient (ADC) measurements as a function of mixing time. In study 2, we compare how well the AXR and CCXR models describe these data, in the presence of crusher gradient induced biases. In study 3, we assess the repeatability of exchange rates estimated using the two models. Finally, in study 4, we test the sensitivity of the AXR and CCXR models to BBB alterations caused by *S. pneumoniae* lung infection and validate these findings against ex-vivo BBB markers. We find that an increase in BBB water permeability during infection is associated with higher levels of plasma von Willebrand factor, a marker of vascular inflammation, and that infection leads to higher levels of astrocytic AQP4 which may drive the observed increases in BBB water exchange.

## Materials and methods

### FEXI sequence and theory

FEXI is double diffusion-encoding sequence which consists of two pulsed gradient spin echo (PGSE) blocks (filter block and detection block), separated by a mixing block, and followed by an echo-planar imaging (EPI) readout (see Fig. 1a). The filter block consists of bipolar diffusion gradients with a fixed filter b-value (defined by gradient pulse amplitude (*g*_f_), pulse duration (*δ*_f_) and interval between onset of the pulses (*∆*_f_)) to attenuate the signal from the fast-diffusing spins. Here, the filter b-value is set to diphase spins in the intravascular compartment. A mixing block follows, where a 90° pulse is applied for longitudinal storage of the magnetisation during a variable mixing time (*t*_m_), allowing time for exchange of spins between intravascular and extravascular compartments. The mixing block either includes a pair of crusher gradients before the second 90° pulse and after the third 90° pulse or uses phase cycling to ensure the correct coherence pathways are selected. Crusher gradients are typically used in imaging experiments (c.f. spectroscopy) instead of phase cycling as there is no requirement to acquire multiple repeats, shortening acquisition time. A spoiler gradient between the two storage pulses is applied to null unwanted transverse magnetisation created by the second 90° pulse. The magnitude of the crusher gradients (*g*_c_) is determined by the slice thickness (∆*z*) and are set at the minimum required dephasing magnitude: *q*_min_ = $\frac{4\pi + \pi \Delta f{\delta }_{s}}{\Delta z}$, where ∆*f* is the radiofrequency (RF) spectral bandwidth and *δ*_s_ is slice gradient duration associated with the 90° storage pulses [43, 44]. The detection block can use different readout b-values (by modulating the gradient pulse amplitude (*g*), pulse duration (*δ*) or interval between onset of the pulses (∆)) for estimation of ADCs.

Since the intravascular and extravascular diffusivities are expected to be *D*_i_ = 6.5 × 10^–3^ mm^2^/s and *D*_e_ = 0.65 × 10^–3^ mm^2^/s respectively [45], the present study targets exchange across the BBB by using low filter b-values (250 s/mm^2^), [27], which attenuates the signal from intravascular spins while leaving extravascular spins mostly unaffected. We aim to quantify the effect of the crusher gradient amplitude (*g*_c_), on the measured ADC as a function of mixing time (ADC(*t*_m_)), and the corresponding impact on the estimated exchange rate. A full derivation of the signal model can be found in: [34, 35]*.* Here, we will describe the standard AXR model followed by the CCXR model.

### The apparent exchange rate (AXR) model

Water exchange across a membrane or barrier can be described using a parameter called the apparent exchange rate (AXR). The AXR model ignores the effects of crusher gradients, it assumes equal relaxation rates between compartments and is only valid in the limit b → 0. Under these conditions, the signal (*S*) can be modelled as:

1 $$S(b,t_{\text{m}} ) = S^{\prime}(t_{\text{m}} )\exp ( - b\, \cdot {\text{ADC}}^{\prime}(t_{{\text{m}}} )),$$

where ${\text{S}}^{\prime}( {t_{{\text{m}}}})$ is the perturbed relaxation weighted signal, $ADC^{\prime}(t_{m} )$ is the filtered ADC measured at mixing time *t*_m_, given by

2 $${\text{ADC}}^{\prime}(t_{m} ) = {\text{ADC}}^{{\text{eq}}} (1 - \,{\sigma}\exp( - {\text{AXR}}. t_{m} )).$$

The non-filtered ADC of the system, ${\text{ADC}}^{{{\text{eq}}}}$ is given by.

3 $${\text{ADC}}^{\text{eq}} = f_{\text{i}}^{\text{eq}} D_{\text{i}} + f_{\text{e}}^{eq} D_{\text{e}} ,$$

and the filter efficiency is

4 $$\sigma = 1 - \frac{{{\text{ADC}}^{\prime}(t_{\text{m}} = 0)}}{{{\text{ADC}}^{\text{eq}}}} = 1 - \frac{f_{\text{e}} (t_{\text{m}} = 0)D_{\text{e}} + f_{\text{i}} (t_{\text{m}} = 0)D_{\text{i}}}{f_{\text{e}}^{eq} D_{\text{i}} + f_{\text{i}}^{eq} D_{\text{i}}},$$

Where $f_{{\text{i}}} \left( {t_{{\text{m}}} } \right)$ and ${f}_{\mathrm{e}}\left({t}_{\mathrm{m}}\right)$ are the visible signal fractions of the “fast” intravascular and “slow” extravascular compartments respectively, and *D*_i_ and *D*_e_ are the intravascular and extravascular diffusivities respectively. The AXR is the apparent exchange rate which can be considered a surrogate index of BBB water permeability. For a two-site system, AXR = *k* = *k*_in_ + *k*_out_ where k_in_/k_out_ are the forward and backward exchange rates respectively.

### Two-compartment crusher compensated exchange rate (CCXR) model

Let us consider the effects of the crusher gradients on the two-compartment signal model. The MRI signal ($\mathbf{S})$ from the slow and fast diffusing compartments can be modelled as a product of matrix exponentials, which describes how the signal evolves through the filter block, mixing block, and diffusion blocks. The dephasing magnitudes (*q*_f,_ *q*_m_ and *q*_d_ from the filter block, mixing block (which incorporates the crusher gradients contribution) and detection block respectively) determine the signal evolution given by [43]:

5 $${\mathbf{S}} = S^{\prime}\left( {t_{{\text{m}}} } \right)e^{{ - \left( {\left( {q_{{\text{f}}}^{2} {\mathbf{D}} + {\mathbf{K}}} \right)t_{{\text{f}}} } \right)}} \times { }e^{{ - \left( {\left( {q_{{\text{m}}}^{2} {\mathbf{D}} + {\mathbf{K}}} \right)t_{{\text{m}}} } \right)}} \times e^{{ - \left( {\left( {q_{{\text{d}}}^{2} {\mathbf{D}} + {\mathbf{K}}} \right)t_{{\text{d}}} } \right)}} {\varvec{f}} ,$$

where $S^{\prime}\left( {t_{{\text{m}}} } \right)$ is the relaxation weighted signal without diffusion encoding; filter b-value, *b*_f_ = *q*_f_^2^*t*_f_, with filter diffusion time, *t*_f_ = *∆*_*f*_*–δ*_*f*_*/*3, mixing block dephasing parameter, r${q}_{\mathrm{m}} = \gamma {g}_{\mathrm{c}}{\delta }_{\mathrm{c}}+ \frac{\gamma {g}_{s}{t}_{s}}{2}$, γ is the gyromagnetic ratio, *g*_c_ is the crusher gradient amplitude and *δ*_c_ is the crusher gradient duration, *g*_s_ is the slice gradient amplitude and *t*_*s*_ is the slice gradient duration and mixing time *t*_m_; detection b value* b* = *q*_d_^2^*t*_d_ with detection diffusion time,* t*_d_ = ∆—δ/3 (see Fig. 1), and

6 $$\mathbf{D}=\left(\begin{array}{cc}{D}_{\mathrm{i}}& 0\\ 0& {D}_{\mathrm{e}}\end{array}\right),$$

where *D*_i_ and *D*_e_ are the diffusion coefficients of the intravascular (fast) and extravascular (slow) compartments respectively, and

7 $$\mathbf{K}=\left(\begin{array}{cc}{k}_{\mathrm{in}}& -{k}_{\mathrm{out}}\\ {-k}_{\mathrm{in}}& {k}_{\mathrm{out}}\end{array}\right),$$

where *k*_in_ and *k*_out_ are the forward/backward exchange rate constants respectively fulfilling the equilibrium condition:

8 $${\mathbf{Kf}} = 0,$$

and

9 $${\mathbf{f}} = \, \left[ {f_{{\text{i}}} ,{ 1} - f_{{\text{i}}} } \right],$$

is a vector of the relaxation weighted signal fractions of the intravascular and extravascular compartments (see Fig. 1b). Here, we assume that relaxation rates in the intravascular and extravascular compartment are equal; further relaxation terms can be incorporated into the model to account for relaxation effects [28, 46].

In addition to providing estimates of water-exchange that are compensated for the impact of crusher gradients, the CCXR enables the two exchange rate contributions to AXR to be individually estimated, providing enhanced physiological specificity. The AXR = *k*_in_ + *k*_out_. Assuming conservation of mass, *k*_in_*f*_i_ = *k*_out_(1-f_i_) and AXR = *k*_in_(1 + *f*_i_/(1*-f*_i_)). Hence the AXR is dependent on both *k*_in_ and the blood volume, *f*_i_, and is therefore not a “pure” exchange rate parameter.

### Animals

Experimental procedures were approved by the Preclinical Imaging Executive Committee of the University of Manchester and carried out in accordance with the UK Animals (Scientific Procedures) Act 1986 and EU Directive 2010/63/EU for animal experiments. Housing and husbandry details conform to the ARRIVE guidelines [47]. Two male and two female wild-type (WT) Fischer and TgF344-AD rats with the APP_swe_ and PS1_Δe9_ mutations were purchased from the laboratory of Prof T. Town (University of Southern California) and were set up as breeding pairs, housed in the Biological Services Unit at the University of Manchester. Genotyping was outsourced to Transnetyx® with only WT animals used in the present study. All animals were housed in groups of 2–4 per cage with individual ventilation, environmental enrichment, constant access to food and water and a 12:12 h cycle of light and dark for the whole duration of the study.

Fifteen male F344 rats, aged 10 ± 3 months, were scanned in total, across the 4 studies in separate sessions: (1) effect of the crusher gradient on ADC’(*t*_m_) and ADC^eq^(*t*_m_) (n = 6); (2) comparison of AXR and CCXR models for BBB water exchange estimation at various slice thicknesses (n = 6); (3) intrasession repeatability for test and retest data collected in the same scan session (n = 15), and (4) effect of mild *Streptococcus pneumoniae* lung infection on BBB for paired baseline and infection data (n = 15). A further set of male F344 rats, aged 8 ± 2 months, were used as non-infected controls (n = 7) for the immunohistochemistry measures in the lung infection study.

### MRI

For each MRI scan, animals were induced with 4% isoflurane anaesthesia and maintained under 2.5% isoflurane mixed into 100% O_2_ at 0.7 L/min. Animals were secured into the MRI cradle with a nose cone, ear bars and a bite bar to minimise head movement. Core body temperature was monitored using a rectal probe (SA Instruments) and maintained at 36.5 ± 0.5 °C using a feedback-controlled hot air blower.

Imaging data were acquired on a Bruker Avance III console (max. gradient strength = 375 mT/m; max. slew rate: 3375 T/m/s) interfaced with an Agilent 7 T 16-cm bore magnet. A Bruker transmit-only resonator (T11070V3) was used for transmission and a two channel Bruker rat brain surface coil (T11205V3) was used for signal reception. An anatomical reference scan was acquired using a T2-TurboRARE sequence for positioning of the slice. For each of the studies, MRI data analysis was performed using Matlab R2021a (Mathworks Inc.).

### *Study 1: Effect of the crusher gradients on ADC’ and ADC*^*eq*^* with mixing time*

We evaluate the impact of the longitudinal storage crusher gradients on the recovery of the filtered and unfiltered apparent diffusion coefficients (ADC’ and ADC^eq^) measurements as a function of mixing time using simulations and in vivo data. The effect of the crusher gradients at different slice thicknesses at low b-value (250 s/mm^2^) was investigated to determine the potential bias in BBB AXR estimates in the rat brain.

### Simulations

To investigate the impact of crusher gradients on ADC'(*t*_m_) and ADC^eq^(*t*_m_), synthetic signals relating to a FEXI experiment incorporating the effects of crusher gradients were generated in Matlab R2021a using a two-compartment model (Eq. [5]) as described previously [43]. The input parameters were: *k*_in_ = 2.38 s^−1^, *k* = *k*_in_ + *k*_out_ = AXR = 2.5 s^−1^ [48], intravascular volume fraction *f*_i_ = 0.05, intravascular and extravascular diffusivities, *D*_i_ = 6.5 × 10^–3^ mm^2^/s and *D*_e_ = 0.65 × 10^–3^ mm^2^/s respectively [45]. Signals were simulated with the filter block switched on and switched off (*b*_f_ = 250 and 0 s/mm^2^ respectively) at five mixing times, *t*_m_ = 0.025, 0.05, 0.1, 0.2 and 0.3 s. The detection block was simulated with eight readout b-values, *b* = 0, 25, 54, 116, 250, 539, 1160, 2500 s/mm^2^.

The simulation above was repeated 3 times with crusher gradients corresponding to slice thicknesses of ∆z = 10.0 mm, 4.0 mm and 2.5 mm. For each slice thickness, the crusher gradient magnitudes were set to the minimum value possible such that spins in the slice direction experienced a dephasing magnitude of 4π [43, 44]:

$$q_\mathrm{m }=\frac{4\pi + \pi \Delta f{\delta }_\text{s}}{\Delta z}$$

where bandwidth ∆*f* = 2000 Hz and slice gradient duration δ_s_ = 1 ms. For each slice thickness, ADC^eq^(*t*_m_) and ADC’(*t*_m_) values were calculated by fitting Eq. [2] to the signal vs b-value data. ADC’(*t*_m_) and ADC^eq^(*t*_m_) values were normalised to ADC^eq^(*t*_m_ = 0).

### In vivo *validation*

To confirm that the simulated effects of crusher gradients on ADC’, ADC^eq^ and AXR are observed in vivo, F344 rats (n = 6) were scanned at slice thicknesses of 2.5 mm and 4.0 mm. The order of the acquisitions for the two slice thicknesses was alternated across the six animals to eliminate potential bias associated with scan duration.

Data were acquired with the filter block switched on and switched off, providing estimates of ADC^eq^(*t*_m_) and ADC’(*t*_m_) at each mixing time. The following imaging parameters were used, which, where possible, were matched to those used to generate the synthetic data described above: filter b-values *b*_f_ = 0, 250 s/mm^2^ with *∆*_f_ = 10 ms; *δ*_f_ = 4 ms, TE_f_ = 16.5 ms; detection block b-values *b* = 0, 250 s/mm^2^ with *∆* = 10 ms; *δ* = 4 ms, crushers gradients applied along the Z-axis with amplitude *g*_c_ = 12.5 and 7.7 mT/m for ∆z = 2.5 and 4.0 mm respectively, duration *t*_c_ = 1.5 ms at bandwidth = 2000 Hz, mixing times, *t*_m_ = 0.025, 0.05, 0.1, 0.2, 0.3 s; and mixing spoiler with amplitude = 40.2 mT/m and duration = 1 ms (corresponding to Fig. 1a). Images were encoded with spin-echo EPI: readout direction = LR, TE = 35.5 ms, TR = 5000 s, single slice with ∆z = 2.5 mm and 4.0 mm, matrix size = 64 × 64, FOV = 32 × 32 mm^2^, resolution = 0.5 × 0.5 × 2.5/4.0 mm^2^ and repetitions = 10. The diffusion gradients were applied in three orthogonal directions (XY, YZ, XZ) for both the filter and detection blocks. The first 90° excitation pulse and 180° refocussing pulses were non-selective with slab thickness = 30 mm. The second and third 90° pulses and the second 180° refocussing pulse were slice selective.

Signals were averaged over diffusion encoding directions using the geometric mean. Mean ADC^eq^ maps (averaged across all repetitions) were generated using the non-filtered data (*b*_f_ = 0 s/mm^2^) acquired at the shortest *t*_m_ (0.025 s) and used to create a binary mask across the whole brain to select values in the range of normal brain tissue 0.65 × 10^–3^ to 1.0 × 10^–3^ mm^2^/s, thereby eliminating voxels contaminated with CSF. The binary mask was used to extract ROI averaged ADC'(*t*_m_) and ADC^eq^(*t*_m_) curves for each animal. ADC'(*t*_m_) and ADC^eq^(*t*_m_) values for each animal were then normalised to their respective ADC^eq^(*t*_m_ = 0.025 s) and curves averaged across all the animals. In a similar fashion to the simulated data, Eq. [2] (normalised to the measured ADC^eq^(*t*_m_ = 0.025 s)) was fit to the normalised ADC'(*t*_m_) curves at each slice thickness. The fitting was constrained for filter efficiency (σ) between 0.0 and 1.0 and AXR between 0.0 and 10.0 s^−1^. The data from one animal was eliminated from the final analysis as the core body temperature unexpectedly dropped during the image acquisition.

### *Study 2: Comparison of AXR and CCXR models for BBB water-exchange estimation at different slice thicknesses*

To investigate whether the CCXR model provides a more accurate fit to ADC'(*t*_m_) data, we fit both AXR and CCXR models to the synthetic and in vivo data acquired in study 1 and compared the fit quality of each model using the Akaike Information Criterion (AIC).

For the AXR model the data was analysed as described in study 1, yielding estimates of AXR and σ. For the CCXR model, Eq. [5] was fit with *D*_i_, *f*_i_, and *k*_in_ as free parameters. To reduce the number of free parameters and stabilise the fit, *D*_e_ was set equal to (ADC^eq^—*f*_i_ x *D*_i_)/(1-*f*_i_) using the fast-exchange assumption as *b* → 0. Unlike the AXR model, which directly models ADC'(*t*_m_), an extra step is needed for the CCXR model fit to first convert modelled signal to modelled ADC'(*t*_m_). Candidate parameters (presented in Table 1) are used to generate modelled signal-vs-*b* data at each mixing time. This is achieved by using Eq. [5] and considering the evolution of the signal passing through the filter block followed by the mixing block and finally through the detection block*.* ADC'(*t*_m_) values are then estimated by finding the gradient of the log of the modelled signal vs b-value data. The CCXR model is fit by finding the candidate parameters (*D*_i_, *f*_i_, and *k*_in_) that minimise the sum of squared differences between the estimated ADC'(*t*_m_) values and the measured ADC'(*t*_m_) values using the Levenberg–Marquardt algorithm.

**Table 1:** Candidate parameters used for the CCXR model

Candidate parameters:	
Mixing time (t_m_)	0.025, 0.05, 0.1, 0.2, 0.3 s
Filter b-value (*b*_f_)	250 s/mm^2^
Detection b-values (*b*)	0, 250 s/mm^2^
Slice thickness (∆*z*)	2.5, 4.0 mm
Gyromagnetic ratio (γ)	2.67 × 10^8^ s^−1^.T^−1^
Crusher gradient amplitude (*g*_*c*_*)*	12.5, 7.7 mT/m
Duration of crusher gradient (*δ*_c_)	1.5 ms
Slice gradient amplitude (*g*_*s*_*)*	3.0, 1.8 mT/m
Duration of slice gradient (*δ*_s_)	1.0 ms
Diffusion time (*t*_d_ = ∆–δ/3)	9.5 ms

Both models were fit to the mean normalised ADC’(*t*_m_) across all animals. Model fits were compared using the AIC = 2 × n_p_ + n_d_ x log(SSE) where n_p_ is the number of model parameters, n_d_ is the number of data points and SSE is the sum of squared differences between the measured and modelled data points.

### *Study 3: BBB-FEXI intrasession repeatability*

To assess the reliability both the AXR and CCXR models, we evaluate the intrasession repeatability at a slice thickness of 4 mm in rats (n = 15). Data were acquired using the same parameters as described above, except ADC^eq^ was collected only at the shortest mixing time (*t*_m_ = 0.025 s). The initial five repetitions and the last five repetitions from the BBB-FEXI acquisition were analysed separately providing two intrasession values of AXR and *k*_in_ for assessment of test–retest repeatability.

A Bland–Altman comparison was performed on the test–retest data to estimate the within-subject standard deviation (${s}_{\mathrm{w}})$ with ${s}_{\mathrm{w}}^{2}= \frac{1}{2\mathrm{n}}\sum {\mathrm{d}}_{\mathrm{i}}^{2}$ where d_i_ is the difference between the two observations for subject i and n is the number of subjects. The 95% agreement limit was calculated at $1.96 {s}_{\mathrm{w}}$. The coefficient of variation (CoV) is given by $\frac{{s}_{\mathrm{w}}}{\upmu } \times 100\%$ to determine the extent of the variability of both methods.

### *Study 4: Validation of BBB-FEXI using mild Streptococcus Pneumoniae lung infection*

To assess the effects of peripheral infection on BBB water permeability, F334 rats (n = 15) were scanned before infection (baseline) and again on day 8–9 of an ascending *Streptococcus pneumoniae* lung infection challenge. The AXR and CCXR models were fit to the mean normalised ADC’(*t*_m_) to obtain estimates of AXR, filter efficiency (σ), *k*_in_, *f*_i_ and *D*_*i*_. Model fitting was performed as described in studies 1 and 2. The data from one animal was eliminated from the final analysis as its data points were detected as outliers.

Von Willebrand factor (VWF) levels, a peripheral marker for vascular injury and inflammation were estimated from blood plasma samples, taken on day 9–10 of infection, in a subset of the rats (n = 9), to investigate the association between vascular inflammation and MRI measures of BBB water-exchange. BBB tight junction proteins (claudin-3, claudin-5, occludin and zonula occludens-1 (ZO1)) and astrocytic water channel protein, AQP4 were assessed by immunohistochemistry staining on day 9 or 10 of infection, in the same subset of rats (n = 9). A further set of non-infected F344 rats (n = 7) were used as age-matched controls, to investigate the BBB pathology induced by infection. Full method details for the *Streptococcus pneumoniae* lung infection protocol, VWF levels and tight junction proteins and AQP4 immunohistochemistry staining can be found in the Supporting Information.

### Statistical analysis

Statistical analysis was performed on GraphPad Prism 9.4.1. A paired two-tailed t-test was used to test the null hypotheses of no difference in AXR and *k*_in_ between baseline and active infection. A Pearson’s correlation was used to determine the relationship between AXR and *k*_in_ during infection against VWF, and the correlation coefficient (r) is used to determine the strength and the direction of the relationship. An unpaired two-tailed student’s t-test was used to compare the mean percent total area of claudin-3, claudin-5, occludin and ZO1 between non-infected and infected animals. An unpaired two-tailed student’s t-test was used to compare AQP4 area under the curve (AUC) values between non-infected and infected animals. p < 0.05 was considered statistically significant in all comparisons.

## Results

### Study 1: Effects of crusher gradients on ADC' and ADC^eq^ with mixing time

**Fig. 2:**
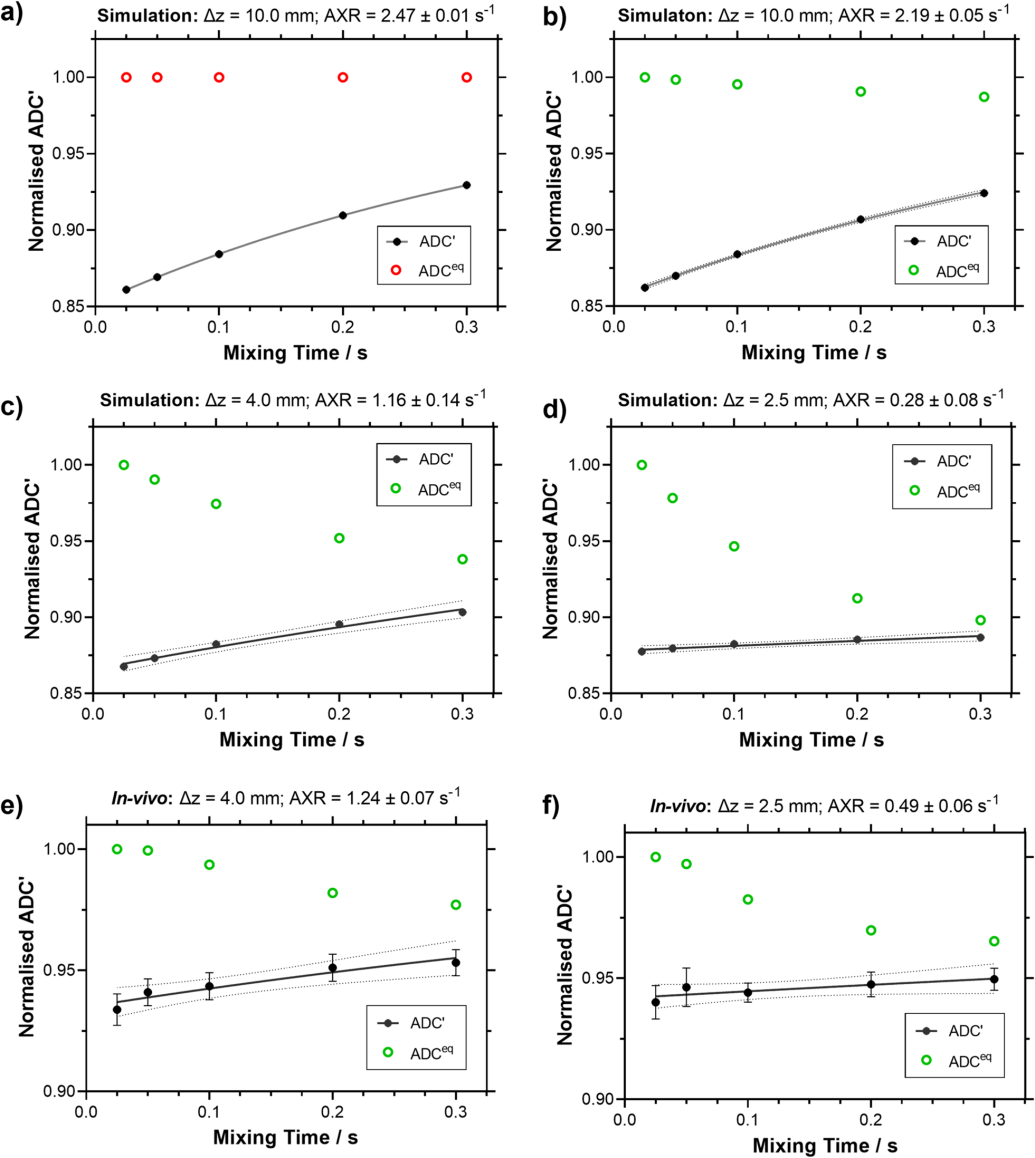
Simulated and in vivo normalised ADC^eq^(*t*_m_) and ADC’(*t*_m_) at a range of slice thicknesses (∆z). **a** Simulated data with crushers off at ∆*z* = 10.0 mm showing the hypothetical ADC'(*t*_m_) and ADC^eq^(*t*_m_) (red circles) behaviour without crusher gradients. Thinner slice thicknesses result in larger decreases in measured ADC^eq^(*t*_m_) (green circles), and more attenuated recovery of ADC'(*t*_m_) (black dots), due to the larger crusher gradients. **b**–**d** Simulated data with active crushers on at ∆*z* = 10.0 mm, 4.0 mm, 2.5 mm. **e, f** In vivo data at ∆*z* = 4.0 mm and 2.5 mm. The AXR model (black solid line) was fit to mean normalised ADC’ with the mean ± standard error (s.e.m) across all animals (n = 5) with 95% confidence displayed (black dash). Different y-axis ranges are used for the normalised ADC’ for the simulated and in vivo data

Figure 2a shows the numerically simulated recovery of the ADC’ against mixing time without the contribution of the crusher gradient at slice thickness, ∆*z* = 10.0 mm, leading to measured AXR of 2.47 s^−1^, in line with the ground truth AXR (2.5 s^−1^). With crusher gradients applied (Fig. 2b–d), the simulations show that as slice thickness decreases, recovery of ADC’(*t*_m_) becomes progressively attenuated leading to underestimation of AXR (2.19 s^−1^, 1.16 s^−1^ to 0.28 s^−1^) for slice thicknesses of 10.0 mm, 4.0 mm to 2.5 mm respectively. The in vivo data is also consistent with increasing AXR underestimation as slice thickness decreases. At slice thicknesses of 4.0 mm and 2.5 mm, estimates of AXR are 1.24 ± 0.07 s^−1^ and 0.49 ± 0.08 s^−1^ respectively (Fig. 2e, f).

The ADC’ recovery will asymptotically tend towards the equilibrium ADC (ADC^eq^) value as *t*_m_ → ∞ (Fig. 2). The underestimation of the AXR can be attributed to the increased dephasing contribution (*q*_m_) caused by the larger crusher gradient magnitude needed for complete dephasing as slice thickness decreases. ADC^eq^ decreases progressively in both simulated and in vivo data as the slice thickness decreases; data presented in the Additional file 1: Table S2. Additionally, there is a decrease in the filter efficiency, as the slice thickness decreases, for both the simulated and in vivo data; Additional file 1: Table S1.

### Study 2: Comparison of AXR and CCXR models for estimating BBB water-exchange at different slice thicknesses

**Fig. 3:**
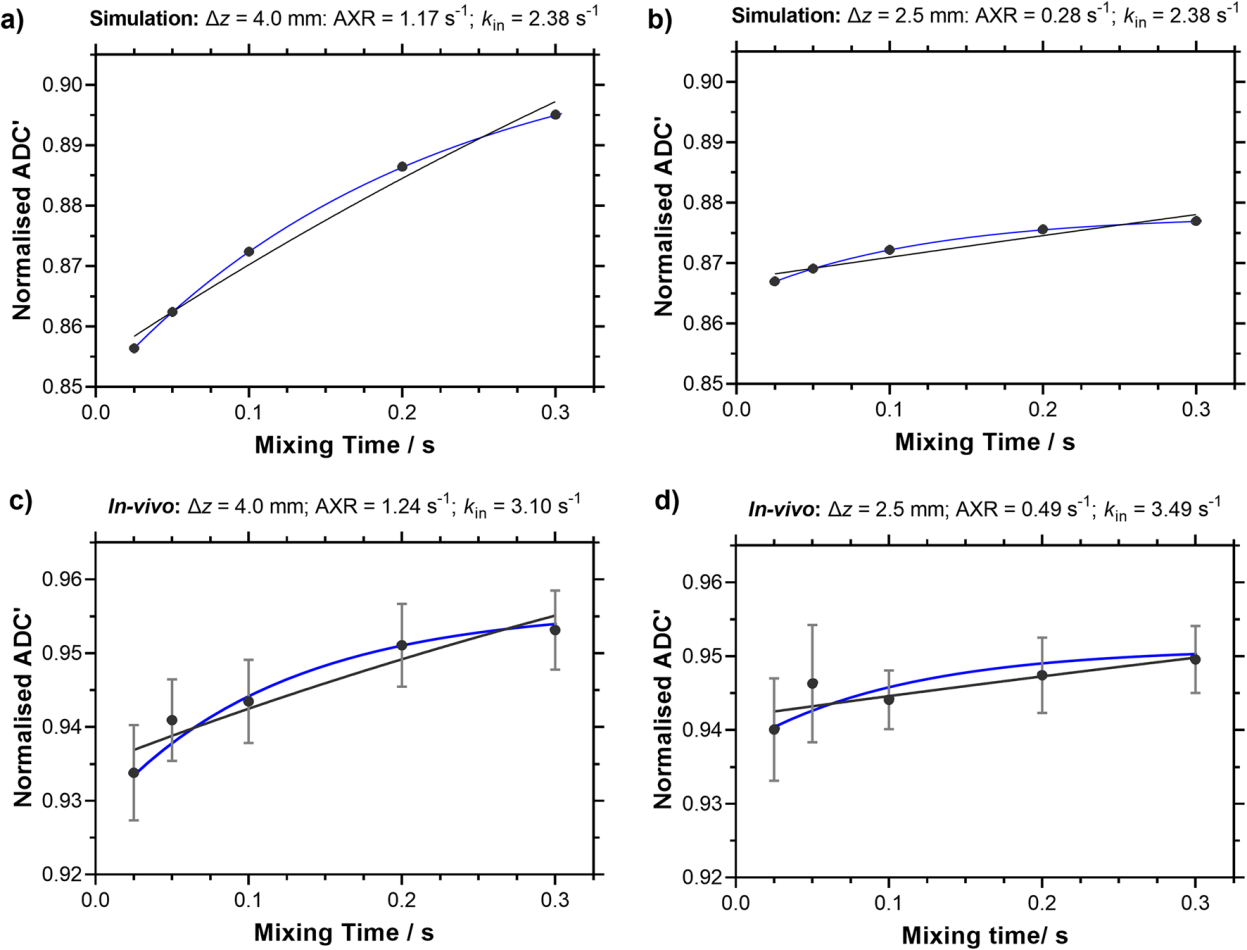
AXR and CCXR models fit to simulated and in vivo ADC’(*t*_m_) at slice thicknesses of 4.0 mm and 2.5 mm. The AXR model (black line) and the CCXR model (blue line) fit to mean normalised ADC’ against mixing time for **a**, **b** simulated data, and **c**, **d** in vivo data. Plots show the mean normalised ADC’ values ± s.e.m across all animals (n = 5). Different y-axis ranges are used for the normalised ADC’ for the simulated and in vivo data

A comparison of the AXR and the CCXR model fits are presented in Fig. 3. The CCXR model was able to recover the ground truth *k*_in_ of 2.38 s^−1^ for the signals simulated at both slice thicknesses at 4.0 mm and 2.5 mm, as expected, compared to the variable AXR estimates of 1.17 s^−1^ and 0.28 s^−1^ when using the AXR model. In the experimental protocol, the CCXR model gave *k*_in_ values of 3.10 s^−1^ and 3.49 s^−1^ for ∆*z* = 4.0 mm and 2.5 mm respectively, which demonstrated the consistency of the model in estimating the exchange rate across different slice thicknesses within the same animals, compared to the AXR estimates of 1.24 s^−1^ and 0.49 s^−1^ respectively (Fig. 3c, d). Furthermore, the CCXR model led to lower values of AIC relative to the AXR model for ∆z = 4.0 mm (− 51.4 vs − 47.5 respectively), although AIC values were similar at ∆z = 2.5 mm for CCXR and AXR models (− 47.6 vs − 49.2 respectively).

### Study 3: BBB-FEXI intrasession repeatability

**Fig. 4:**
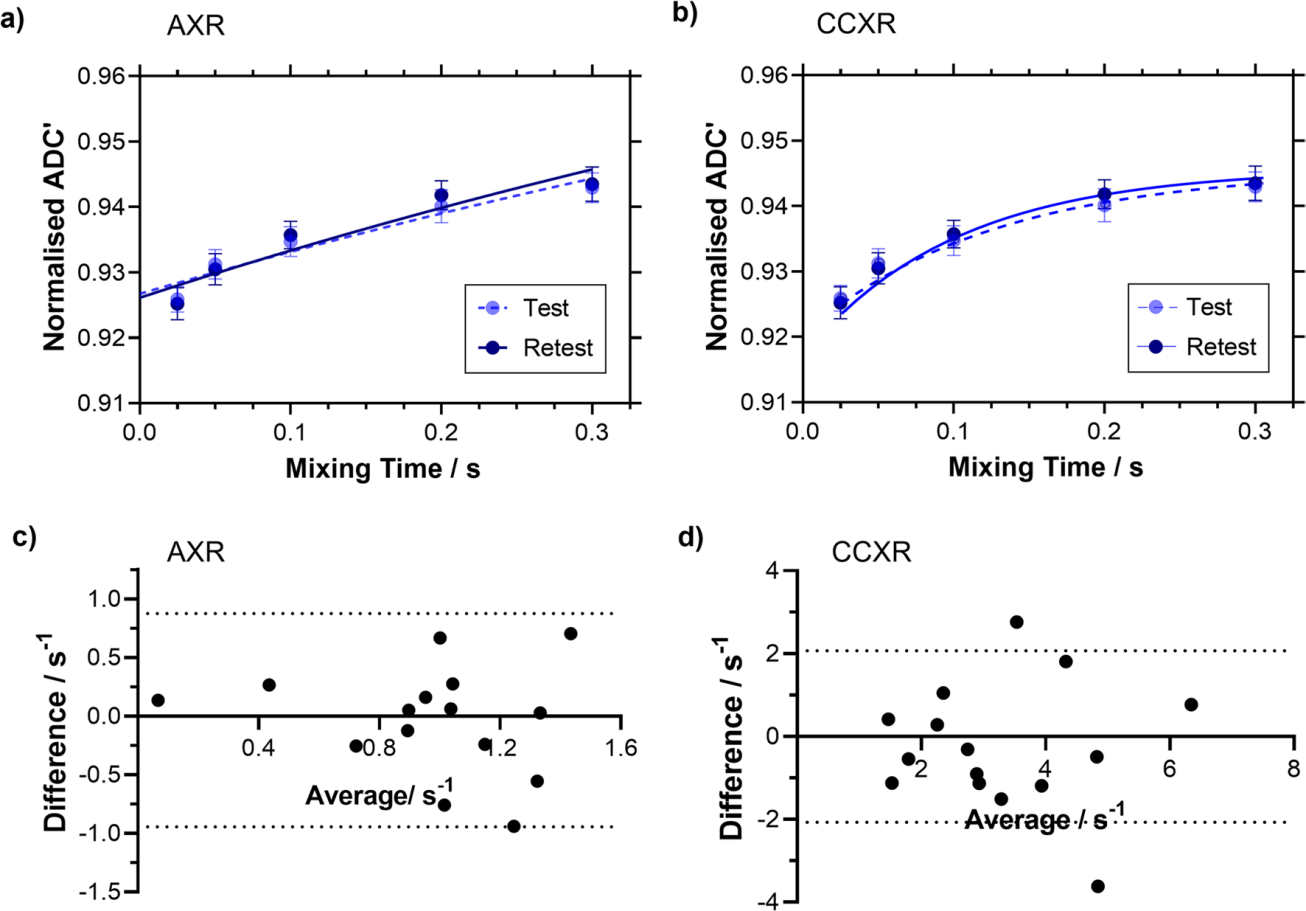
Intrasession Repeatability Mean normalised apparent diffusion coefficient (ADC’) against mixing times for the test and retest with mean ± s.e.m displayed for **a** the apparent exchange rate (AXR) model fit (n = 15) and **b** the crusher-compensated exchange rate (CCXR) model fit (n = 15). Bland–Altman plots of the **c** average vs the difference in AXR estimates (n = 15) and **d** average vs the difference in *k*_in_ for each pair of test—retest measurements with the black dotted line showing 95% limits of agreement

The mean BBB water exchange values for the test and retest acquisitions were AXR = 0.97 ± 0.02 s^−1^ and *k*_in_ = 3.19 ± 0.07 s^−1^ (n = 15). Bland-Altmann plots show the spread in test and retest AXR and *k*_in_ measurements, and the 95% limits of agreement (Fig. 4c, d). The CoV is 33% for the AXR model and 32% for the CCXR model. The values for ADC^eq^, filter efficiency, intravascular diffusivity and intravascular signal fraction for the test and retest scans are presented in Additional file 1: Figure S1.

### Study 4: Validation of BBB-FEXI using mild *Streptococcus Pneumoniae* lung infection

**Fig. 5:**
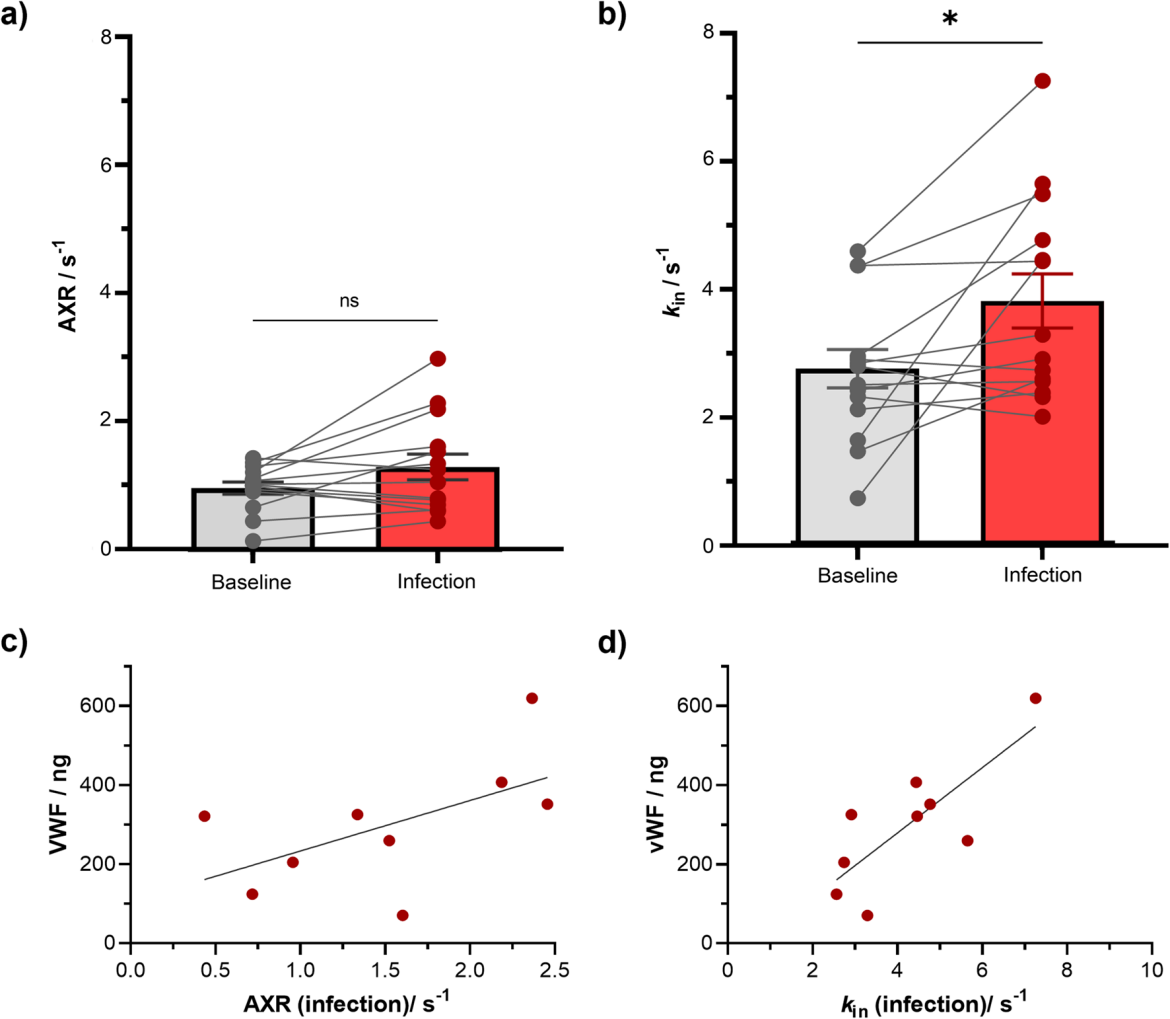
BBB water exchange increase and vascular inflammation from infection **a** Apparent exchange rate (AXR) estimates for individual animals (n = 14) from the AXR model at baseline and following infection with *S. Pneumoniae*, ns: non-significant **b** BBB water exchange (*k*_in_) measurements in individual animals (n = 14) from using CCXR model at baseline and following infection with *S. Pneumoniae*. BBB water exchange measurements against the concentration in von Willebrand factor (VWF) in plasma samples of individual rats (n = 9) for **c** AXR during infection; p = 0.11, r = 0.57 and **d** *k*_in_ during infection; *p = 0.01, r = 0.79

The rats showed a varied response to the *S. pneumoniae* lung infection which is reflected in the range of BBB water exchange values (AXR and *k*_in_) following infection (Fig. 5a, b). The AXR values increased from 0.96 ± 0.09 s^−1^ (baseline) to 1.29 ± 0.20 s^−1^ following *S. pneumoniae* infection, but AXR change did not reach significance; p = 0.07 (Fig. 5a). However, we observe a significant 70 ± 10% increase in the forward exchange rate (*k*_in_) from baseline (2.72 ± 0.30 s^−1^) to infection (3.78 ± 0.42 s^−1^) obtained using the CCXR model; p = 0.02, Fig. 5b. There was no significant correlation between AXR values during infection and plasma VWF (p = 0.11; r = 0.57, Fig. 5c), but a significant positive correlation was observed for *k*_in_ (p = 0.01; r = 0.79, Fig. 5d).

Representative ADC’ maps for a representative animal at baseline and during infection can be found in Additional file 1: Figure S2a. Mean normalised ADC’(*t*_m_) plots, at baseline and during infection, fit to both the AXR and CCXR models are also presented in Additional file 1: Figure S2c-d. There was a strong correlation between AXR and *k*_in_ values (Additional file 1: Figure S2b). No significant differences were measured in the filter efficiency (σ) or the equilibrium apparent diffusion coefficient (ADC^eq^(*t*_m_ = 0.025 s)) between baseline and infection; data presented in Supplementary Information (Additional file 1: Figure S3a-b). Similarly, there were no significant differences between the intravascular diffusivities (*D*_i_) or intravascular signal fraction (*f*_i_) between baseline and infection (Additional file 1: Figure S3c-d).

**Fig. 6:**
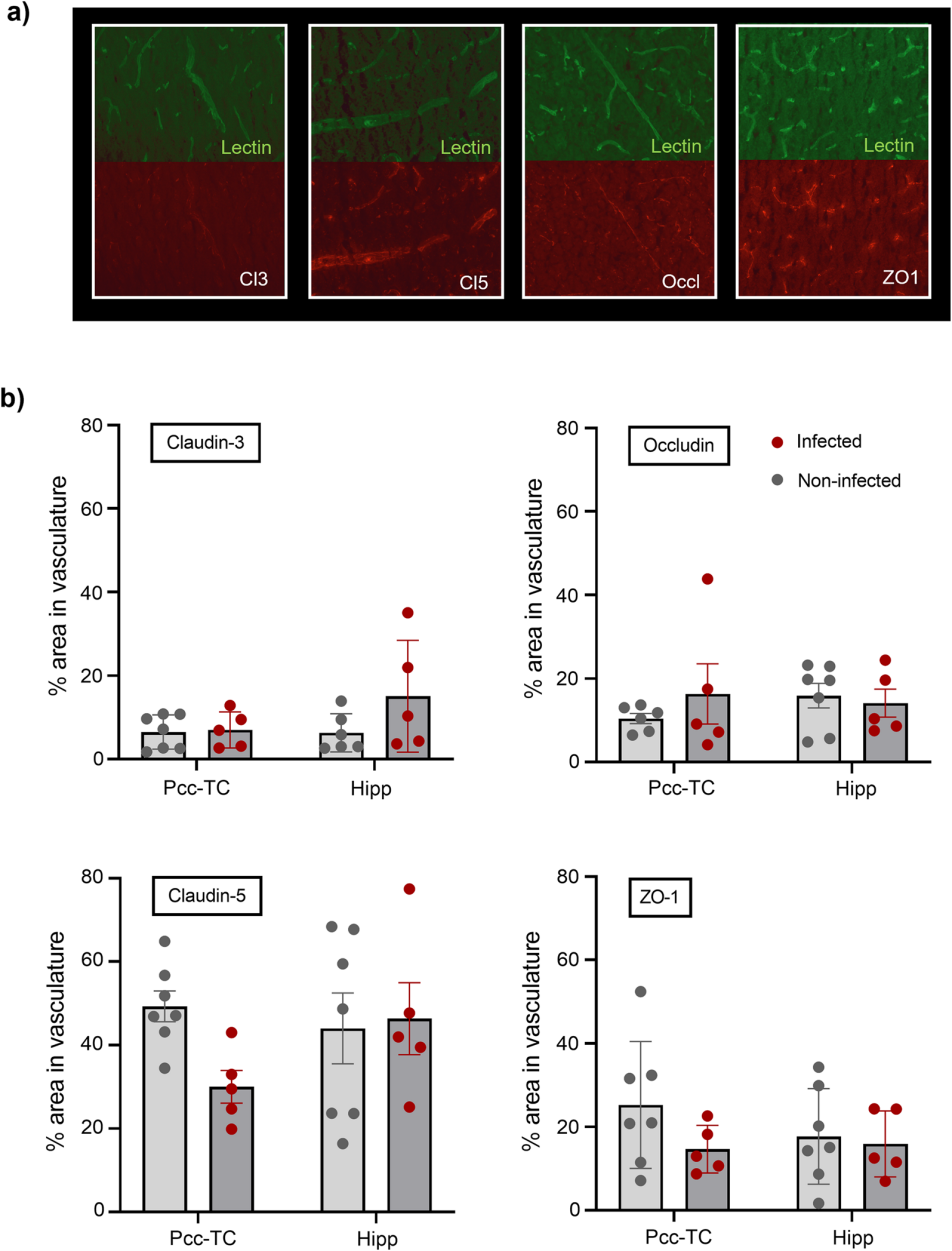
Expression of tight junction proteins in non-infected and infected animals **a** Representative immunofluorescence images from the posterior cingulate ad temporal cortices region in non-infected animals of lectin and tight junction proteins claudin-3 (Cl3), claudin-5 (Cl5), occludin (Occl) and zonula occludens-1 (ZO1). **b** Percentage (%) area of the image covered by tight junction proteins in vasculature for posterior cingulate and temporal cortex (Pcc-TC) and hippocampus (Hipp) for non-infected (n = 7) and infected rats (n = 5). Plot shows individual animal data with the mean ± s.e.m displayed

No significant changes were measured in any of the tight junction protein markers when comparing the posterior cingulate and temporal cortices and hippocampal brain regions of non-infected to infected animals (Fig. 6b). This would suggest that the tight junctions remain intact following our mild lung infection protocol. Additional file 1: Table S3 in the Supporting Information provides the values of percentage area in the vasculature covered by each tight junction protein.

**Fig. 7:**
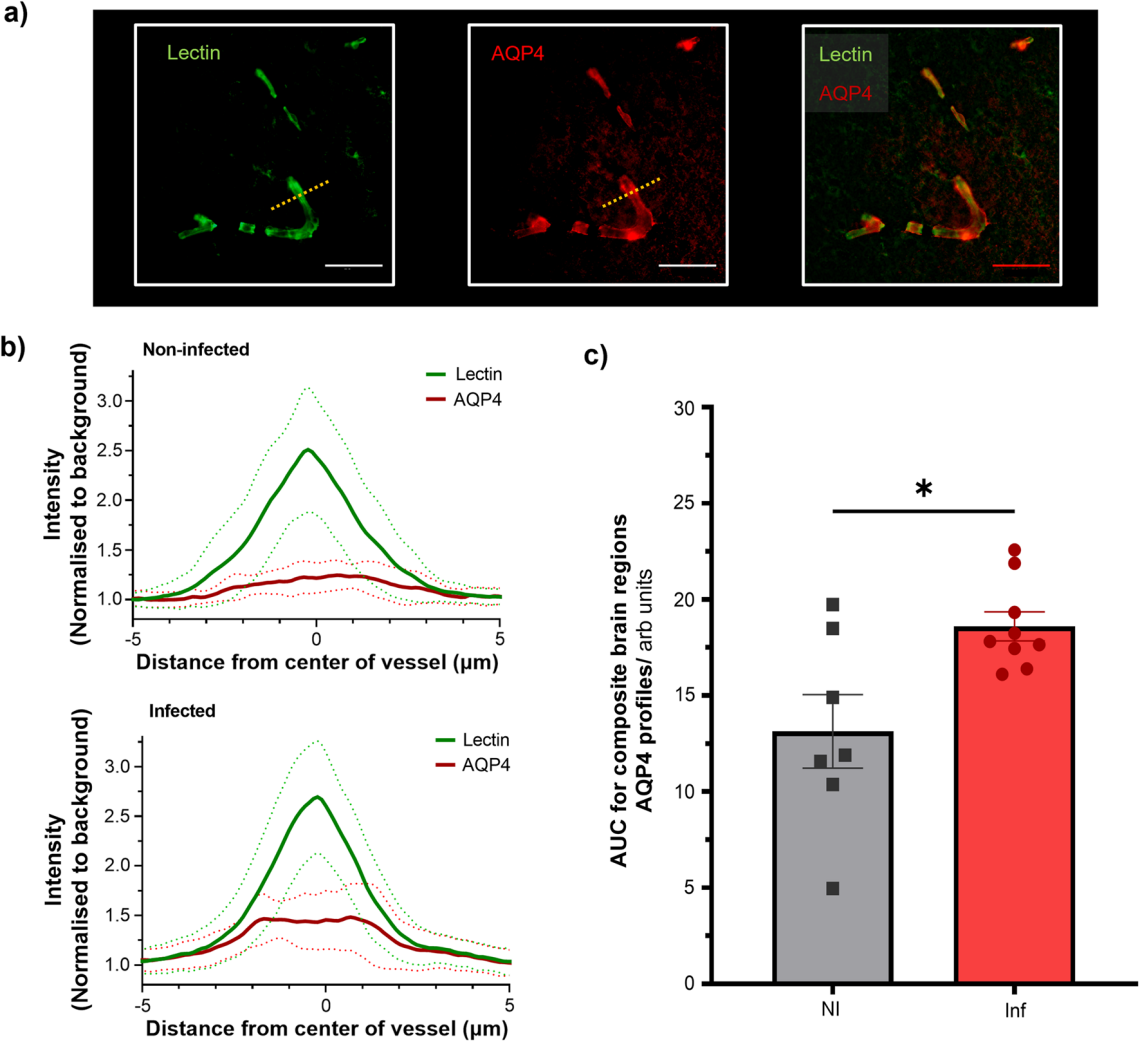
Aquaporin-4 (AQP4) water channel association with infection. **a** Representative immunofluorescence images from an infected animal showing lectin (left), AQP4 (middle) and composite image of both the lectin and AQP4 staining (right), scale bars indicate 50 μm. The yellow dashed line indicates a typical line profile used to measure the intensity profiles as shown in **b** mean intensity profiles for the lectin (green) and AQP4 (red) from a representative non-infected animal (top) and a representative infected animal (bottom) taken from the single brain region. **c)** Mean area under curve (AUC) measurements from the individual AQP4 profiles averaged across all brain regions for non-infected (NI) animals (n = 7) and infected (Inf) animals (n = 9). Plot shows mean AUC values ± s.e.m; * p = 0.01

Lectin and AQP4 intensity profiles for representative non-infected and infected animal are shown in Fig. 7b. We observed a significantly higher AUC in AQP4 intensity profiles of infected animals (42% higher) relative to non-infected animals (18.6 ± 0.8 vs 13.1 ± 1.9 arb units) (p = 0.01, Fig. 7b). The AUC in lectin profiles remained consistent between the infected and non-infected groups, results presented in (Additional file 1: Figure S5b).

## Discussion

We have developed a blood–brain barrier filter-exchange imaging (BBB-FEXI) technique to assess water permeability across the BBB and have shown in the rat brain that it is sensitive to BBB alterations caused by *Streptococcus pneumoniae* lung infection. We demonstrate that the apparent exchange rate (AXR) model leads to underestimation of the BBB water exchange in the presence of longitudinal storage crusher gradients, particularly with smaller slice thickness (2.5 mm). To address this, we propose a more complete crusher compensated exchange rate (CCXR) model which accounts for additional diffusion weighting caused by the crusher imaging gradients and removes the associated bias in estimated exchange rates. We show through simulations that the CCXR model was able to recover accurate exchange rate (*k*_in_) values independent of slice thickness, and CCXR estimates from in vivo data agreed more favourably with existing literature data on BBB water permeability measured using MRI. Both AXR and CCXR models estimated BBB water exchange with reasonable test–retest repeatability. A significant 70 ± 10% increase in *k*_in_ was measured in rats following a mild *S. pneumoniae* lung infection. BBB water exchange during the infection estimated using the CCXR model showed a strong correlation with plasma VWF levels, a marker of vascular inflammation. While BBB tight junction protein markers remained unchanged, we found that the perivascular expression of the astrocytic water channel protein (AQP4) was 42% higher in infected animals compared to non-infected controls, which could plausibly drive the increases in water exchange observed during infection. Our results demonstrate that the BBB-FEXI technique is sensitive to BBB alterations caused by peripheral infection and could be a promising tool for better understanding neuroinflammation and BBB processes in disease.

The implementation of the BBB-FEXI method in the rat brain is challenging due to the low filter b-value (250 s/mm^2^) required to target water exchange across the BBB and the thinner slices required for the markedly smaller size rodent brain. Lasič et al*.* suggest 2.5 mm as the minimum slice thickness for a negligible AXR bias; however higher filter b-values (1000 s/mm^2^) were used in their study, which focussed on measuring transcellular water exchange [43]. In the present study, which aims to measure BBB exchange instead of transcellular exchange, lower filter b-values are needed to attenuate the signal from the faster moving spins in the intravascular compartment, which exacerbates the underestimation of AXR due to the relatively larger contribution of crusher gradients to the low-pass diffusion filter. Synthetic data, simulated using a two-compartment exchange model, show a ninefold underestimation of BBB water exchange measurements when using the AXR model at ∆*z* = 2.5 mm. Our experimental data also reflects this marked decrease in AXR estimates with decreasing slice thickness (Fig. 2e-f). The AXR model does not account for the additional diffusion weighting due to the crusher gradients, but the use of thicker slices reduce this effect. The differences between the simulated and experimental data (predominantly in the filter efficiency, σ, Additional file 1: Table S2) may arise from lower intravascular signal fraction (*f*_i_) in the rat brain compared to when simulated (*f*_i_ = 0.05), which is supported by *f*_i_ estimates (0.019 ± 0.002) from repeatability study in (Additional file 1: Figure S1d). Our results demonstrate that ∆*z* = 2.5 mm is not currently feasible for obtaining accurate BBB water exchange measurements using the AXR model.

To address the bias introduced by crusher gradients on exchange rate estimates, we have proposed a new model for BBB-FEXI measurements that can account for the effects of crusher gradients. The CCXR model allows accurate estimates of water exchange rates to be recovered despite the bias in ADC’(*t*_m_) imposed by the crusher gradients. In simulated data, the CCXR model removes the bias in *k*_in_ induced by the crusher gradient (comparing Fig. 3a, b, c and d) and is able to recover exchange rates that are relatively independent of slice thickness for in vivo data (*k*_in_ at 3.10 s^−1^ and 3.49 s^−1^ for ∆*z* of 4.0 mm and 2.5 mm respectively). Importantly, this model provides measurements of water exchange in the rodent brain that are more consistent with the literature estimates (~ 2.5 s^−1^) [48] derived from measurements of BBB water permeability using a range of techniques. The difference in AIC (− 3.9) between the CCXR and the AXR models at ∆z = 4.0 mm would suggest that CCXR provides an improved fit compared to the AXR model, though at ∆z = 2.5 mm the two fits are more comparable. The CCXR model could be implemented for the analysis of clinical data at small slice thicknesses for both BBB and for transcellular membrane water exchange. The technique demonstrated good repeatability at slice thickness of 4.0 mm for BBB water exchange estimates in the rat brain, with CoV of 33% and 32% for AXR and *k*_in_ respectively.

To determine whether our BBB-FEXI technique was sensitive to BBB pathology we conducted experiments in rats before and during mild lung infection with *S. pneumoniae*. Peripheral infection, which induces a systemic inflammatory response, is known to affect BBB function [6, 49]. Imaging studies have been able to detect BBB dysfunction caused by widespread inflammation from lupus [50, 51], but to our knowledge, BBB alterations due to lung infection have yet to be studied using non-invasive approaches. Our hypothesis was that *S. pneumoniae* infection would create a subtle modulation of BBB permeability detectable using our measures of BBB water exchange. The *S. pneumoniae* infection protocol used a clinically relevant human isolate which induced a range of responses in the animals (see Fig. 5). Overall, in accordance with our hypothesis, we found a significant increase in *k*_in_ following infection, which to our knowledge is the first non-invasive demonstration of altered BBB water exchange dynamics caused by systemic inflammation. While the CCXR model detected this increase, the AXR model did not.

There is evidence to suggest that *S. pneumoniae* triggers systemic inflammation that induces BBB dysfunction. A previous study demonstrated that exposure to *S. pneumoniae* lung infection worsened BBB damage in rats with induced stroke, measured by the influx of IgG protein into the brain parenchyma [52]. The extent of vascular inflammation caused by lung infection can be measured by the plasma levels of VWF, which is created and stored in endothelial cells and released into the blood following vascular injury. Here, we found the levels of plasma VWF had a strong positive correlation with *k*_in_ during infection, suggesting that peripheral infection could promote BBB water exchange via a vascular inflammatory response, which may be non-disruptive, since no marked changes in levels of tight junction proteins were found. Further investigation could explore other brain-immune cross-talk mechanisms that occur in peripheral infection that could alter water exchange across the BBB.

Du et al. found an increase in astrocytic *Aqp4* gene expression following a peripheral lipopolysaccharide (LPS) challenge which is a common model used to trigger inflammation in animals [53]. We found that infected rats had 42% higher expression of astrocytic AQP4 in the combined hippocampal, posterior cingulate and temporal cortices (Additional file 1: Figure S5a), which facilitates the rapid transfer of water across the BBB [54, 55]. A multi-slice acquisition would allow the effects of infection to be investigated in further brain regions. It has also been previously shown that in *Aqp4* knockout mice, cortical BBB water permeability was significantly reduced using a related MRI technique (multiple-echo-time ASL) [30]. Altogether, these studies provide increasing evidence to support the key role of AQP4 in water permeability across the BBB, particularly in the context of systemic inflammation. AQP4 has been shown to drive neuroinflammation via the release of interleukin-6 [56], which is further evidence to support water exchange as a promising biomarker for neuroinflammatory mechanisms. Components of the BBB may also be altered in different ways if the infection is more severe or sustained over a longer period of time or in presence of other neuropathological alterations such as amyloid deposition and angiopathy in AD.

Future studies should implement a comparison between BBB-FEXI and other BBB water exchange MRI techniques (e.g. arterial spin labelling or advanced contrast enhanced methods). This could help to better understand the precise water transport mechanisms that each technique is probing (such as from the intravascular or perivascular spaces) and help to quantify additional method biases. RF phase cycling could be considered in future studies as an approach to modify the FEXI technique [57], particularly given multiple repetitions are often needed to boost SNR. Accounting for potential relaxation rate differences in the intravascular and extravascular compartments could further increase specificity of the exchange measurements, which have not been examined in the present study, but can be modelled [46] and have recently been implemented using BBB-FEXI in the human brain [28].

Preclinical MRI is fundamentally limited by the small brain size of rodents. To achieve adequate sensitivity with our BBB-FEXI protocol, we need to use the entirety of a fairly thick slice, hence losing the resolution required to measure exchange in small and anatomically relevant brain regions. The CCXR model enables thinner slices to be acquired in both the human and the rodent brain, which could be valuable for probing specific small brain regions particularly affected in diseases, such as the hippocampus in Alzheimer’s disease, or the *substantia nigra* in Parkinson’s disease. The technique could be extended to a multi-slice acquisition in future studies for wider brain coverage. In humans, BBB-FEXI can achieve higher SNR due to larger voxels and is able to provide regional estimates of water exchange rates [28].

## Conclusion

We have established a preclinical blood–brain barrier filter exchange imaging technique that is able to reliably and accurately estimate BBB water exchange. In particular, we propose the new crusher compensated exchange rate analysis to account for the substantial biases in water exchange introduced by the longitudinal storage crusher gradients and allows for accurate BBB water exchange estimates with thinner imaging slices. This non-invasive technique detected increased water exchange across the BBB following a mild *S. pneumoniae* infection, which was associated with higher VWF in blood plasma and higher expression of AQP4 water channels at the BBB. BBB-FEXI is a promising tool for detecting and monitoring early BBB dysfunction in brain diseases, and for understanding the impact of peripheral infection on the BBB.

## Supplementary Information

**Additional file 1: Table S1.** List of antibodies used for the immunohistochemistry experiments. **Table S2.** Modelling parameters: Apparent exchange rate (AXR), equilibrium apparent diffusion coefficient (ADC^eq^) and filter efficiency (σ) at various slice thicknesses (∆*z*) from simulated and in vivo data. **Table S3.** Percentage (%) area of tight junction protein covering the vasculature in each brain region for non-infected and infected animals. **Figure S1.** Modelling parameters from test and retest study: a) Equilibrium apparent diffusion coefficient (ADC^eq^) for test (0.81 ± 0.01 x 10^-3^ mm^2^/s) and retest (0.81 ± 0.01 x 10^-3^ mm^2^/s) scans b) Filter efficiency (*σ*) for test (0.073 ± 0.002) and retest (0.075 ± 0.002) scans c) Intravascular diffusivity (*D*_i_) for test (0.017 ± 0.003 mm^2^/s) and retest (0.020 ± 0.005 mm^2^/s) scans d) Intravascular signal fraction (*f*_i_) for test (0.019 ± 0.002) and retest (0.019 ± 0.002). All plots show individual animal data with mean ± s.e.m across all animals (n = 15); ns: non-significant. **Figure S2.** Apparent diffusion coefficient (ADC’(*t*_m_)) measures for AXR and *k*_in_ estimates at baseline and during infection_._ a) ADC’ maps at each mixing time (*t*_m_*)* from a representative animal at baseline and during infection (voxels where ADC’ > 1.0 x 10^-3^ mm^2^/s have been removed), with T2 TurboRARE anatomical images (left),. b) Plot of individual water exchange rate measures, *k*_in_ against AXR, both at baseline and during infection (n = 28); p < 0.0001, r = 0.76. Mean normalised ADC’ against mixing time across all animals (n = 14) with mean ± s.e.m fit to c) the apparent exchange rate (AXR) model and d) the crusher compensated exchange rate (CCXR) model. **Figure S3.** Modelling parameters from in rats at baseline and during infection: a) Equilibrium apparent diffusion coefficient (ADC^eq^) at baseline (8.16 ± 0.04 x 10^-4^ mm^2^/s) and during infection (8.15 ± 0.03 x 10^-4^ mm^2^/s) b) Filter efficiency (*σ*) at baseline (0.076 ± 0.003) and during infection (0.076 ± 0.002) c) Intravascular diffusivity (*D*_i_) at baseline (0.016 ± 0.003 mm^2^/s) and during infection (0.020 ± 0.004 mm^2^/s) d) Intravascular signal fraction (*f*_i_) at baseline (0.019 ± 0.002) and during infection (0.018 ± 0.002). All plots show individual animal data with mean ± s.e.m displayed across all animals (n = 14); ns: non-significant. **Figure S4: **Immunohistochemistry slices a) Schematic of the top view of the rat brain showing slice locations from bregma, and the sagittal view showing the two brain regions of interest: posterior cingulate and temporal cortices (light orange) and hippocampus (dark orange). b) Representative example of the brain slices from for the tight junction and AQP4 staining, three locations in each brain slice for each brain region were acquired. **Figure S5**. Aquaporin-4 (AQP4) and lectin profiles a) Individual area under curve (AUC) for AQP4 profiles from the hippocampus (Hipp) and posterior cingulate and temporal cortices (Pcc-TC) brain regions. 2-way ANOVA with multiple-comparisons; *p < 0.05. b) Mean AUC measurements from the vessel (lectin) profiles across hippocampal and posterior cingulate and temporal cortices brain regions for non-infected (NI) animals (n = 7) and infected (Inf) animals (n = 9). Plot shows individual animal data with mean ± s.e.m displayed; ns: non-significant.

## Data Availability

The datasets used and analysed during the current study are available from the corresponding author on reasonable request.

## Abbreviations

AD: Alzheimer’s disease
ADC: Apparent diffusion coefficients
ADC’: Filtered apparent diffusion coefficient
ADC^eq^: Unfiltered apparent diffusion coefficient
AIC: Akaike information criteria
ASL: Arterial spin labelling
AXR: Apparent exchange rate
*Aqp4*: Aquaporin-4 gene
AQP4: Aquaporin-4 water channel protein
AUC: Area under the curve
BBB: Blood–brain barrier
CCXR: Compensated exchange rate
CoV: Coefficient of variation
Cl3: Claudin-3
Cl5: Claudin-5
DCE-MRI: Dynamic contrast enhanced magnetic resonance imaging
EPI: Echo-planar imaging
FEXI: Filter-exchange imaging
GBCA: Gadolinium-based contrast agents
LPS: Lipopolysaccharide
MCI: Mild cognitive impairment
NVU: Neurovascular unit
Occl: Occludin
RF: Radiofrequency
PGSE: Pulsed gradient spin echo
*S. pneumoniae*: *Streptococcus pneumoniae*
SNR: Signal-to-noise ratio
VWF: Von Willebrand factor
WEPCAST: Water extraction with phase contrast arterial spin tagging
WT: Wild-type
ZO1: Zonula occludens-1
