# Supplementary material for: Filter exchange imaging with crusher gradient modelling detects increased blood–brain barrier water permeability in response to mild lung infection
Source: Fluids Barriers CNS. 2023 Apr 3;20:25. doi: 10.1186/s12987-023-00422-7 (PMC10071630; doi:10.1186/s12987-023-00422-7)
Supplement: Supplementary file 1 — Additional file 1: Table S1. List of antibodies used for the immunohistochemistry experiments. Table S2. Modelling parameters: Apparent exchange rate (AXR), equilibrium apparent diffusion coefficient (ADCeq) and filter efficiency (σ) at various slice thicknesses (∆z) from simulated and in vivo data. Table S3. Percentage (%) area of tight junction protein covering the vasculature in each brain region for non-infected and infected animals. Figure S1. Modelling parameters from test and retest study: a) Equilibrium apparent diffusion coefficient (ADCeq) for test (0.81 ± 0.01 x 10^-3^ mm2/s) and retest (0.81 ± 0.01 x 10^-3^ mm2/s) scans b) Filter efficiency (σ) for test (0.073 ± 0.002) and retest (0.075 ± 0.002) scans c) Intravascular diffusivity (Di) for test (0.017 ± 0.003 mm2/s) and retest (0.020 ± 0.005 mm2/s) scans d) Intravascular signal fraction (fi) for test (0.019 ± 0.002) and retest (0.019 ± 0.002). All plots show individual animal data with mean ± s.e.m across all animals (n = 15); ns: non-significant. Figure S2. Apparent diffusion coefficient (ADC’(tm)) measures for AXR and kin estimates at baseline and during infection. a) ADC’ maps at each mixing time (tm) from a representative animal at baseline and during infection (voxels where ADC’ > 1.0 x 10^-3^ mm2/s have been removed), with T2 TurboRARE anatomical images (left),. b) Plot of individual water exchange rate measures, kin against AXR, both at baseline and during infection (n = 28); p < 0.0001, r = 0.76. Mean normalised ADC’ against mixing time across all animals (n = 14) with mean ± s.e.m fit to c) the apparent exchange rate (AXR) model and d) the crusher compensated exchange rate (CCXR) model. Figure S3. Modelling parameters from in rats at baseline and during infection: a) Equilibrium apparent diffusion coefficient (ADCeq) at baseline (8.16 ± 0.04 x 10^-4^ mm2/s) and during infection (8.15 ± 0.03 x 10^-4^ mm2/s) b) Filter efficiency (σ) at baseline (0.076 ± 0.003) and during infection (0.076 ± 0.002) c) Intravas [file 12987_2023_422_MOESM1_ESM.docx]

**Additional file**

**Study 4: Validation of BBB-FEXI using mild *Streptococcus Pneumoniae* lung infection Methods**

**Streptococcus Pneumoniae Lung Infection Protocol**

A protocol for doses and timing of *Streptococcus pneumoniae* (American Type Culture Collection [ATCC] 49619, capsular serotype 19F [Danish]) challenge was established to allow low-grade, indolent pulmonary exposure to *S. pneumoniae* infection that is sustained in animals for a period of 9 to 10 days (Dénes, Pradillo et al. 2014).

*S. pneumoniae* bacteria were plated on sheep blood agar (Becton, Dickinson and Company) and incubated for 24h at 37°C with 5% CO_2_. 15-20 colonies were added to 25ml of growth media (brain heart infusion (Becton, Dickinson and Company) supplemented with 20 % of heat-inactivated foetal bovine serum (HIFBS)) which was incubated on an agitator at 37°C in 5% CO_2_ atmosphere until an optical density, measured using a spectrophotometer, of 0.6 (mid-log phase) was reached. The concentration was calculated using the Miles Misra technique (Miles, Misra et al. 1938). These cultures were centrifuged at 9 G and pellets were stored at -80°C as stock inoculates. Stock was washed in sterile PBS, re-suspended and 50 μl was delivered intranasally between the two nostrils in an ascending challenge at concentrations of 4 x10^8^ cfu/ml (day 1), 8 x 10^8^ cfu/ml (day 3) and 16 x10^8^ cfu/ml (day 6). None of the infected rats showed any signs of neurological symptoms during the infection challenge.

**Von Willebrand Factor by ELISA**

Von Willebrand factor (VWF) is a peripheral marker for vascular injury and inflammation. VWF levels was estimated from blood plasma samples, collected 1 day after final infection scans, in a subset of the rats (n = 9).

Ninety-six well plates were coated with 100 µl per well of VWF capture antibody (1:1000 = 3.1 μg/ml; Dako A0082) at 4 ᴼC overnight. For all the immunohistochemistry experiments, PBS refers to Dulbecco′s Phosphate Buffered Saline (without calcium chloride and magnesium chloride, Sigma, at 100 mM). Wells were washed with 3 x 300 ul/well PBS + 0.1% (PBST), then blocked with 200 μl/well PBST + 2.5 % bovine serum albumin (BSA) (Sigma) for 2 hours on a shaker at room temperature. Plasma samples and a standard dilution of normal human plasma (0, 7.8, 15.6, 31.25, 38.5, 62.5, 83.3, 125 ng/ml) were diluted in PBST 1% BSA and measured in duplicates. Samples and standards (100 µL) were added to wells in duplicate and incubated for 2hrs at room temperature on a shaker. Wells were washed with PBST before incubation with 100 µL of VWF peroxidase antibody (1:1000 = 7.33 nM; Dako P0226) in PBST 1% BSA was measured at 492 nm in a plate reader and sample absorbances were converted to VWF concentrations based on interpolation of the standard curve in GraphPad Prism 9.4.0.

**Tight junction and AQP4 staining by immunohistochemistry**

BBB tight junction proteins (claudin-3, claudin-5, occludin and zona-occudens-1) and the water channel protein (AQP4) were assessed *ex-vivo* to investigate potential alterations to the BBB structure in response to the lung infection. The same subset of infected rats described in VWF measurements (n = 9) were culled by transcardiac perfusion with heparinised saline (10 000 units/mL). A further set of non-infected F344 rats (n = 7) were also culled in the same manner and used as age-matched controls. The brains were collected, snap frozen using isopentane on dry ice and stored at -80°C. Sagittal brain sections (20 µm thick) were taken on SuperFrost Plus glass slides using a cryostat (Leica CM3050s, Leica Biosystems Nussloch GmbH, Germany). The brains were cut in the sagittal plane with cryostat (Leica CM3050s, Leica Biosystems Nussloch GmbH, Germany) into 20 µm thick sections at approximately 0.90, 1.46 and 2.02 mm lateral of Bregma and collected on SuperFrost Plus glass slides and stored at -80°C until used.

Slides were defrosted and allowed to dry at room temperature. Sections were fixed for 10 min by incubation in 4% paraformaldehyde in PBS. After 6 × 5 min washes in PBS, the slides were incubated with a permeabilization-blocking solution (0.1% Triton X-100 (Sigma-Aldrich, T9284), 2% of Animal-Free Blocker® (Vector Laboratories, SP-5030-250) in PBS) used for all the incubation steps below. For AQP4 staining, an additional antigen retrieval step was performed prior to blocking (30 minutes in 10 mM sodium citrate buffer, pH = 6, 97°C in a water bath). Autofluorescence was quenched using TrueBlack® (Biotium #23007) according to the manufacturers instructions.

The primary antibody incubation was performed overnight at 4 °C. The following day, sections were washed in PBS 3 times for 10 min and incubated with secondary antibody (Molecular Probes, see Supplementary Table S1) and tomato lectin (1:250; Sigma Aldrich, L0401) for 2 h. For full details about the antibodies, please refer to Supporting Information Table S1. The slides were then washed 3 x 10 min and mounted with ProLong™ Gold Antifade Mounting medium (Thermo-Fisher, P36934). The mounted sections were allowed to dry at room temperature and kept stored at 4 °C until imaging.

All images were acquired at 20× magnification with a fluorescence microscope (Olympus BX51) and QCapturePro 7 ® software. For each animal, two regions of interest were imaged (hippocampus and the posterior cingulate and temporal cortices), and three replicates per region were acquired, see Figure S4. The total percentage area of each tight junction proteins in the vasculature (from the lectin staining) was measured across each of the brain regions.

For AQP4 quantification, line profiles were manually drawn across five vessels in each lectin image, in ImageJ (Fiji). The line profiles were used to extract the intensity profile for both lectin and AQP4 staining. Each profile was then normalised to the background intensity defined as the average intensity at the edge of the profile, to subtract out differences in background intensity. Profiles were then centred using the peak lectin intensity as a reference point. For each animal, lectin and AQP4 profiles were averaged across replicates and across the two regions of interest to generate a composite profile. The mean area under the curve (AUC) of the lectin and AQP4 profiles was calculated for each individual animal.

**Table S1:** List of antibodies used for the immunohistochemistry experiments.

|  | **Primary antibodies** | | | **Secondary antibodies** | |
| --- | --- | --- | --- | --- | --- |
| **Target protein** | **Manufacturer (Reference)** | **Specie raised in** | **Dilution** | **Manufacturer & Reference** | **Dilution** |
| Claudin 3 | Abcam  (ab15102) | rabbit | 1:500 | Molecular Probes -Invitrogen  goat anti-mouse 594 (SAB4600105),  and goat anti-rabbit (SAB4600107) | 1:500 |
| Occludin | ThermoFisher- Invitrogen  (71-1500) | rabbit | 1:500 |  |  |
| Claudin 5 | ThermoFisher- Invitrogen  (35-2500) | mouse | 1:150 |  |  |
| Zona occludens 1 | Thermofisher, (339100 (WA316684)) | mouse | 1:1000 |  |  |
| Aquaporin-4 | Millipore  (AB3594) | rabbit | 1:1000 |  |  |

**Additional Results**

**Table S2: Modelling parameters:** Apparent exchange rate (AXR), equilibrium apparent diffusion coefficient (ADC^eq^) and filter efficiency (σ) at various slice thicknesses (∆*z*) from simulated and *in vivo* data.

**
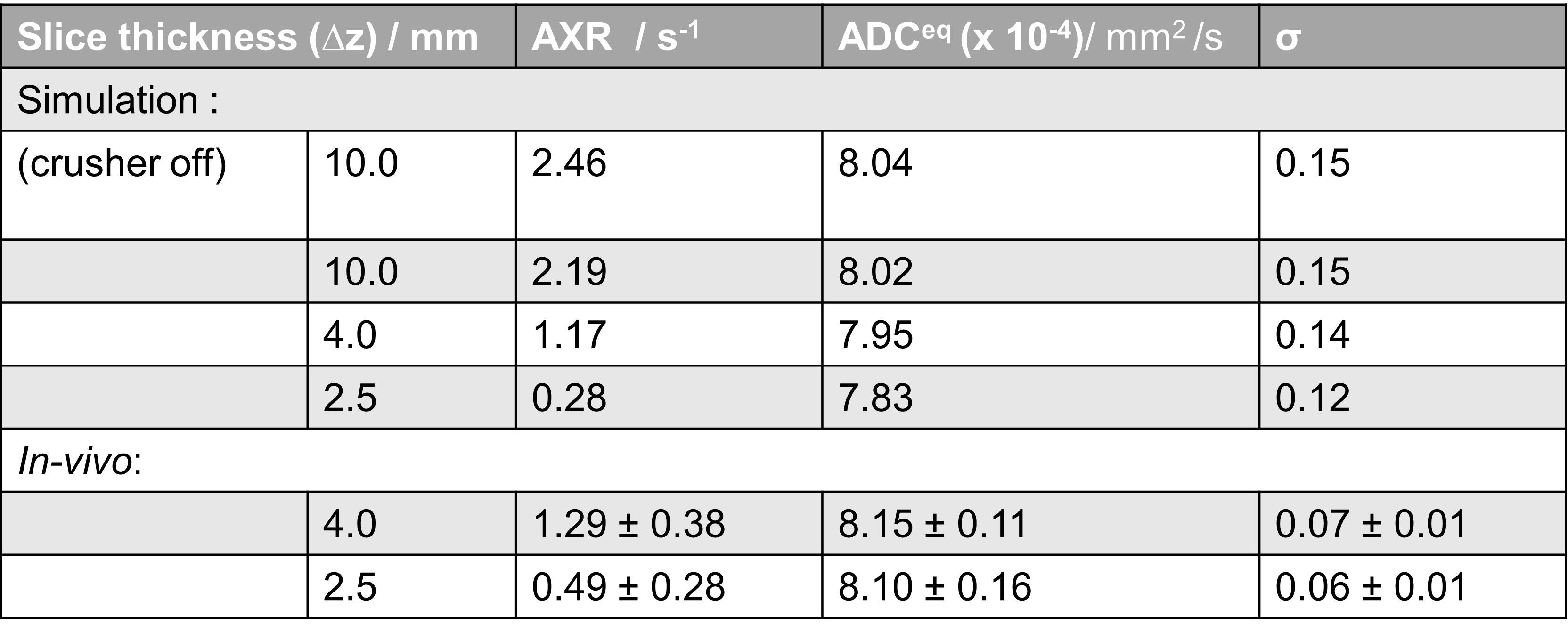
**


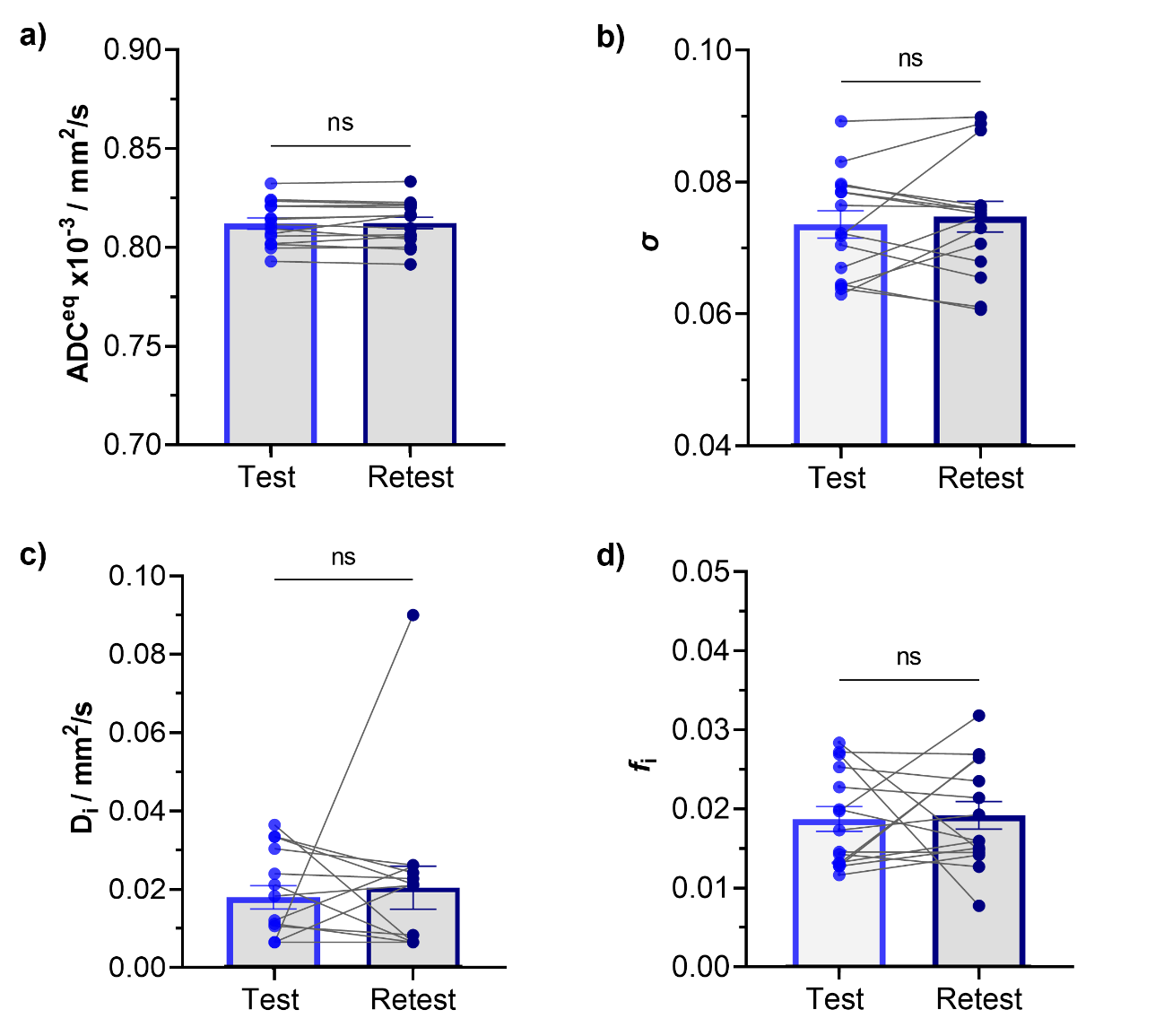


**Figure S1: Modelling parameters from test and retest study: a)** Equilibrium apparent diffusion coefficient (ADC^eq^) for test (0.81 ± 0.01 x 10^-3^ mm^2^/s) and retest (0.81 ± 0.01 x 10^-3^ mm^2^/s) scans **b)** Filter efficiency (*σ*) for test (0.073 ± 0.002) and retest (0.075 ± 0.002) scans **c)** Intravascular diffusivity (*D*_i_) for test (0.017 ± 0.003 mm^2^/s) and retest (0.020 ± 0.005 mm^2^/s) scans **d)** Intravascular signal fraction (*f*_i_) for test (0.019 ± 0.002) and retest (0.019 ± 0.002). All plots show individual animal data with mean ± s.e.m across all animals (n = 15); ns: non-significant.


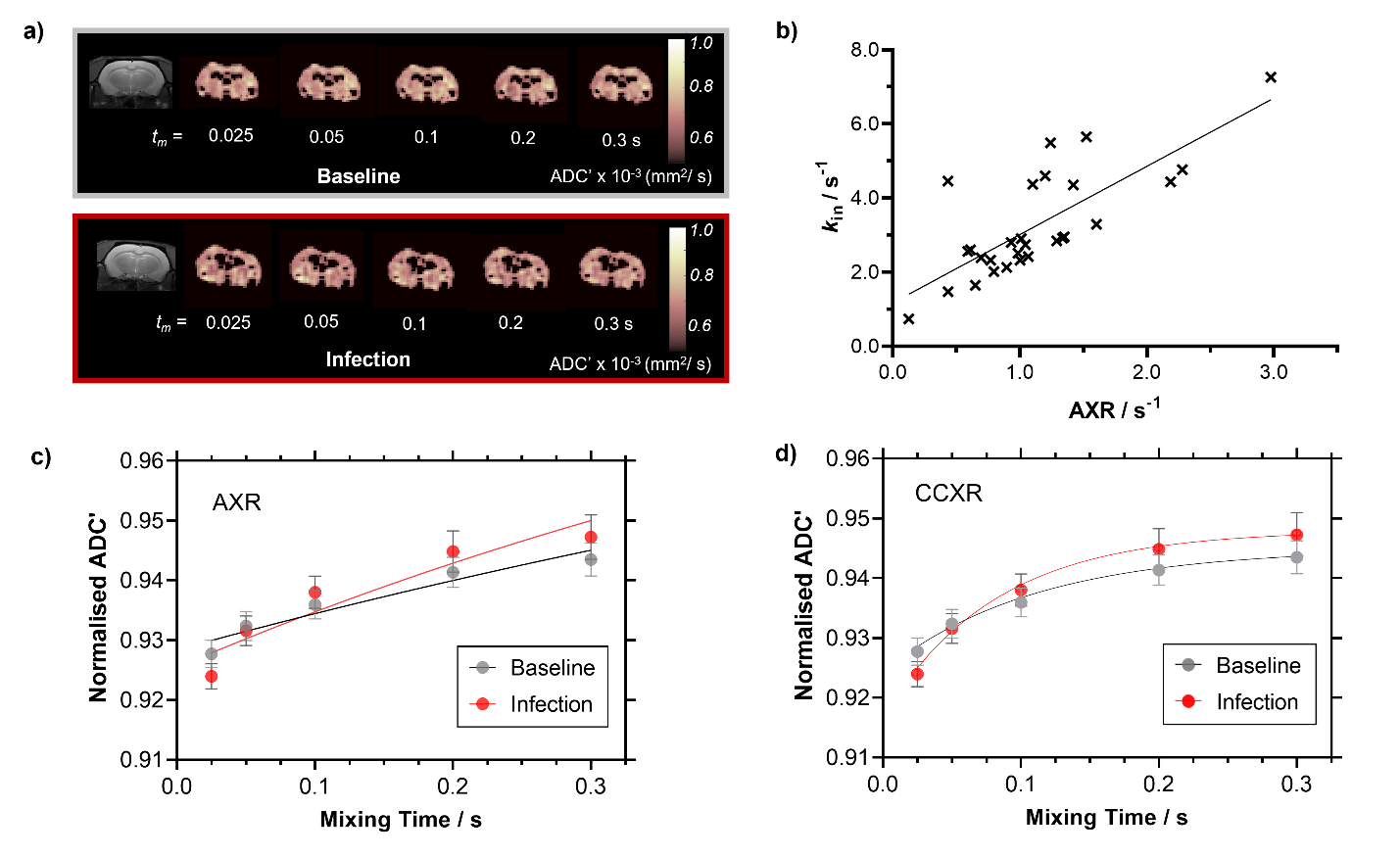


**Figure S2: Apparent diffusion coefficient (ADC’(*t*_m_)) measures for AXR and *k*_in_ estimates at baseline and during infection_._ a)** ADC’ maps at each mixing time (*t*_m_*)* from a representative animal at baseline and during infection (voxels where ADC’ > 1.0 x 10^-3^ mm^2^/s have been removed), with T2 TurboRARE anatomical images (left),. **b)** Plot of individual water exchange rate measures, *k*_in_ against AXR, both at baseline and during infection (n = 28); p < 0.0001, r = 0.76. Mean normalised ADC’ against mixing time across all animals (n = 14) with mean ± s.e.m fit to **c)** the apparent exchange rate (AXR) model and **d)** the crusher compensated exchange rate (CCXR) model.


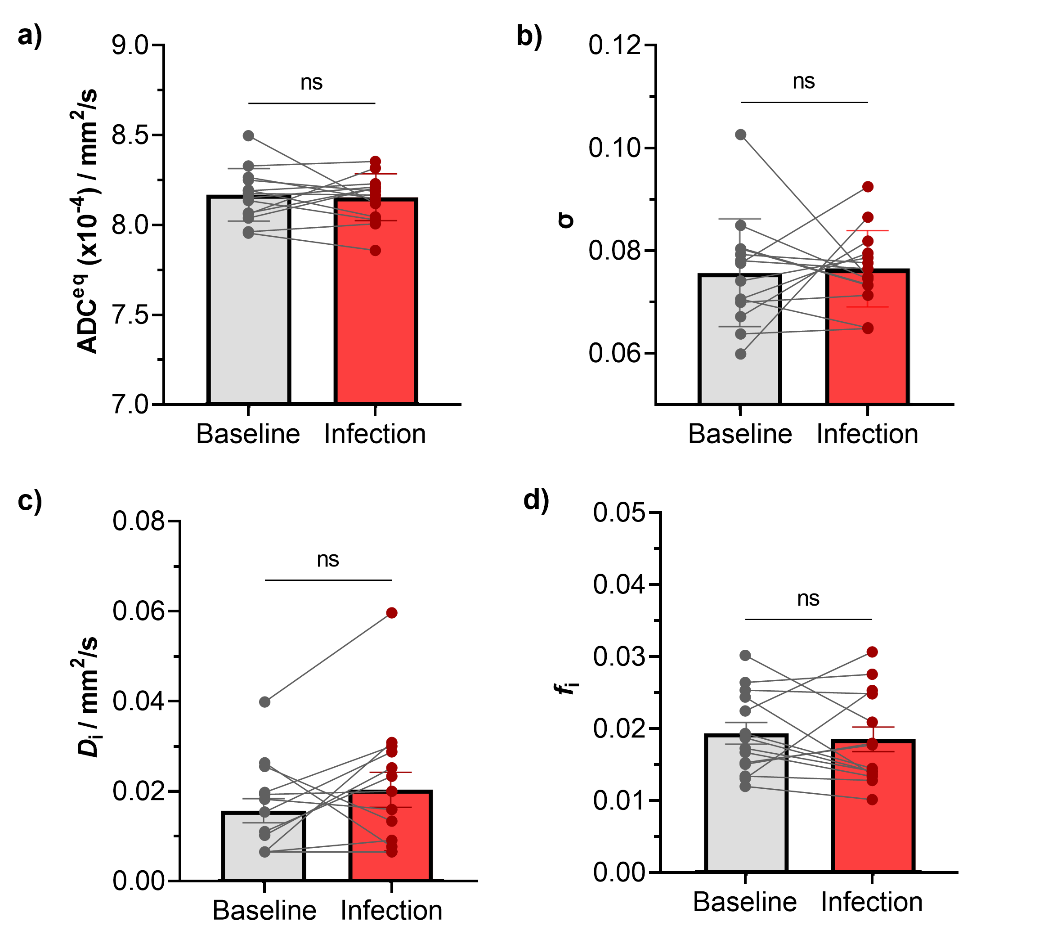


**Figure S3: Modelling parameters from in rats at baseline and during infection: a)** Equilibrium apparent diffusion coefficient (ADC^eq^) at baseline (8.16 ± 0.04 x 10^-4^ mm^2^/s) and during infection (8.15 ± 0.03 x 10^-4^ mm^2^/s) **b)** Filter efficiency (*σ*) at baseline (0.076 ± 0.003) and during infection (0.076 ± 0.002) **c)** Intravascular diffusivity (*D*_i_) at baseline (0.016 ± 0.003 mm^2^/s) and during infection (0.020 ± 0.004 mm^2^/s) **d)** Intravascular signal fraction (*f*_i_) at baseline (0.019 ± 0.002) and during infection (0.018 ± 0.002). All plots show individual animal data with mean ± s.e.m displayed across all animals (n = 14); ns: non-significant.


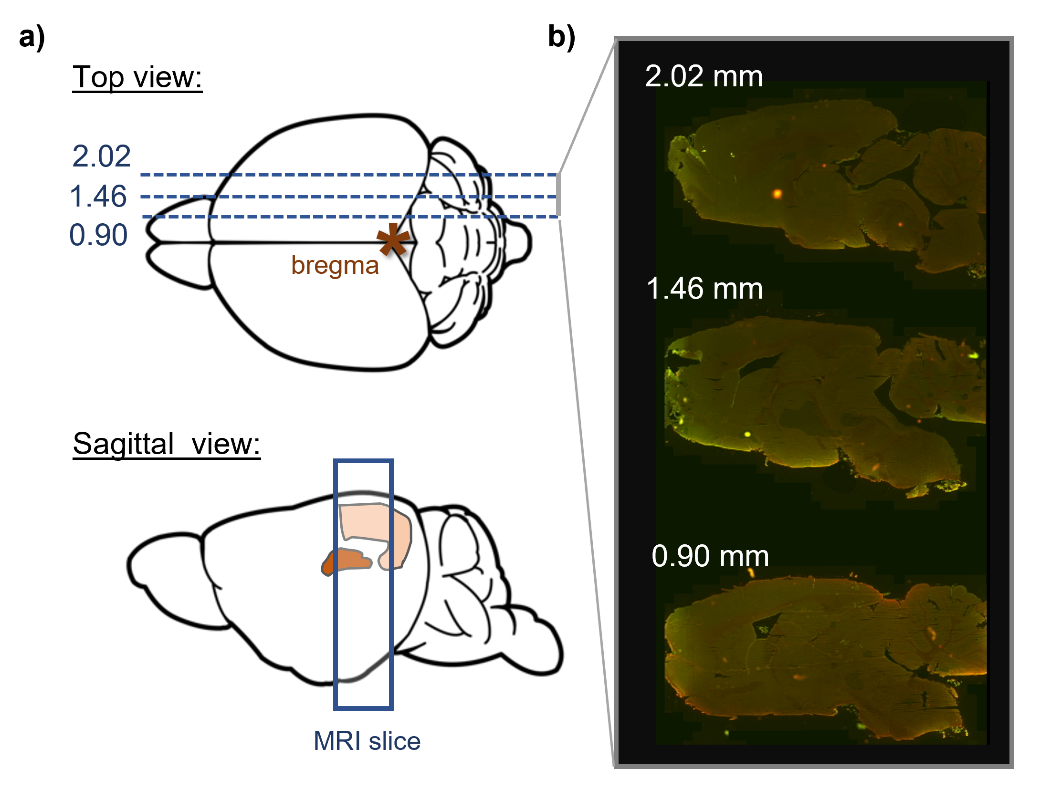


**Figure S4: Immunohistochemistry slices** **a)** Schematic of the top view of the rat brain showing slice locations from bregma, and the sagittal view showing the two brain regions of interest: posterior cingulate and temporal cortices (light orange) and hippocampus (dark orange). **b)** Representative example of the brain slices from for the tight junction and AQP4 staining, three locations in each brain slice for each brain region were acquired.

**Table S3: Percentage (%) area of tight junction protein covering the vasculature in each brain region for non-infected and infected animals**.


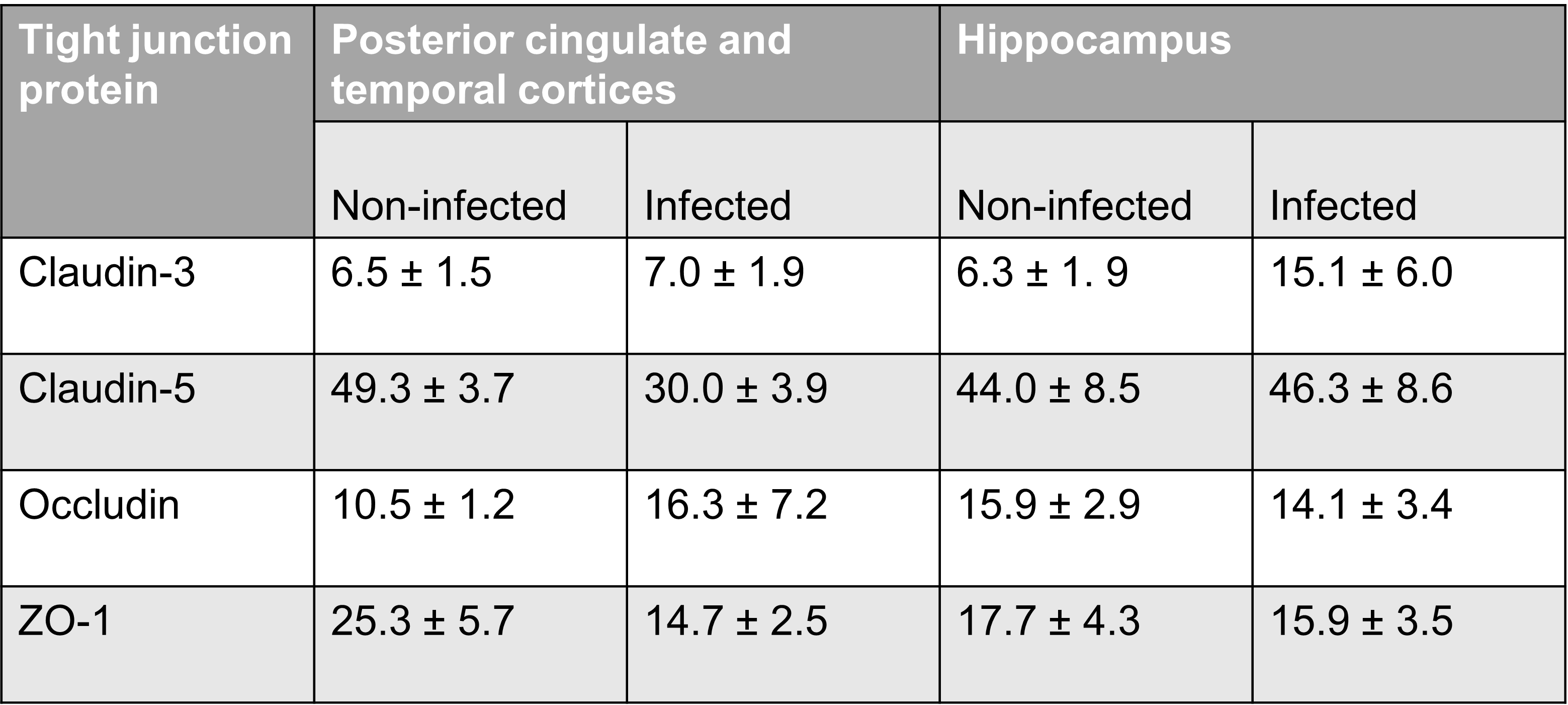


**
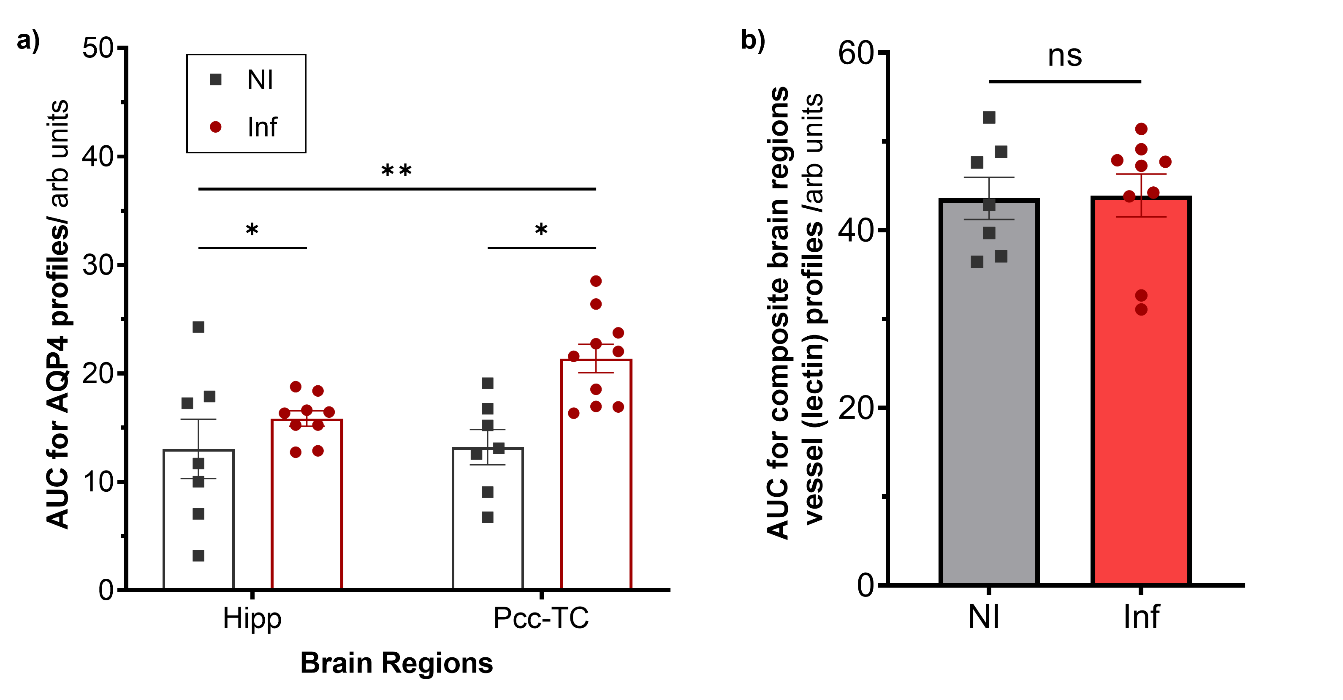
**

**Figure S5: Aquaporin-4 (AQP4) and Lectin profiles** **a)** Individual area under curve (AUC) for AQP4 profiles from the hippocampus (Hipp) and posterior cingulate and temporal cortices (Pcc-TC) brain regions. 2-way ANOVA with multiple-comparisons; *p < 0.05. **b)** Mean AUC measurements from the vessel (lectin) profiles across hippocampal and posterior cingulate and temporal cortices brain regions for non-infected (NI) animals (n = 7) and infected (Inf) animals (n = 9). Plot shows individual animal data with mean ± s.e.m displayed; ns: non-significant.
